# Recent advances in biomaterials for osteosarcoma treatment

**DOI:** 10.1093/rb/rbaf087

**Published:** 2025-08-21

**Authors:** Jian Han, Mingna Huo, Chenxu Jia, Bolun Zhang, Fengping Zhang, Qingtao Meng

**Affiliations:** Department of Orthopedics, Dalian No.3 People’s Hospital, Dalian, Liaoning 116091, China; Department of Orthopedics, Dalian No.3 People’s Hospital, Dalian, Liaoning 116091, China; Department of Orthopedics, Dalian No.3 People’s Hospital, Dalian, Liaoning 116091, China; Department of Orthopedics, Dalian No.3 People’s Hospital, Dalian, Liaoning 116091, China; Department of Orthopedics, Dalian No.3 People’s Hospital, Dalian, Liaoning 116091, China; Department of Orthopedics, Dalian No.3 People’s Hospital, Dalian, Liaoning 116091, China

**Keywords:** osteosarcoma, biomaterials, biomaterial-based delivery systems, bone regeneration, bone repair

## Abstract

Osteosarcoma is a highly aggressive bone malignancy with poor prognosis due to high metastasis and drug resistance. Conventional treatments often result in systemic toxicity and limited efficacy, highlighting the need for more precise and integrative approaches. Biomaterials with excellent biocompatibility and functional tunability have emerged as promising tools to enhance local therapy and support bone regeneration. This review summarizes recent advances in the application of natural, synthetic and composite biomaterials in four key areas: drug delivery, gene therapy, immunotherapy and post-resection bone repair. To provide a systematic perspective, we compiled and classified 64 representative studies published between 2021 and 2025, comparing biomaterial-based delivery strategies ranging from single-material carriers to multifunctional composite platforms. Particular focus is given to stimuli-responsive systems and scaffolds that integrate antitumor activity with regenerative capacity. By outlining emerging strategies and material platforms, this review offers a concise reference for the rational design of biomaterials addressing the dual challenge of tumor eradication and skeletal reconstruction. Interdisciplinary collaboration will be key to advancing these systems toward clinical application.

## Graphical abstract

**Figure:**
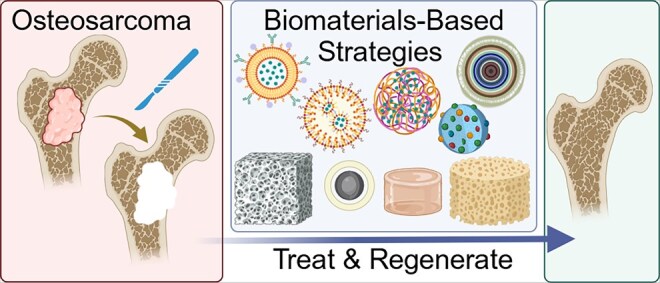

## Introduction

Osteosarcoma is the most common primary malignant bone tumor, characterized by the production of osteoid matrix by neoplastic cells [1]. It predominantly affects children, adolescents and young adults, with a peak incidence during the adolescent growth spurt [2]. Despite its relative rarity, accounting for less than 1% of all cancers diagnosed annually in the United States, osteosarcoma represents a significant challenge in pediatric oncology [1, 3]. The incidence is slightly higher in males than females, with approximately 1000 new cases diagnosed each year in the United States, of which about 500 occur in individuals under 20 years of age [1, 4].

The current standard of care for osteosarcoma involves a multimodal approach combining surgery, systemic chemotherapy, and in some cases, radiation therapy [5]. While this treatment paradigm has dramatically improved outcomes since its introduction, with 5-year survival rates increasing from less than 20% in the 1970s to 60–70% for patients with localized disease, significant challenges remain [1, 6]. For patients presenting with metastatic disease at diagnosis (approximately 15–20%), the prognosis remains poor, with 5-year survival rates as low as 19% [7]. Moreover, 30–40% of patients with initially localized disease will experience recurrence, further highlighting the limitations of current therapeutic approaches [8, 9]. The mainstay of systemic therapy for osteosarcoma relies on a combination of high-dose methotrexate, doxorubicin (DOX) and cisplatin [10]. However, this intensive chemotherapy regimen is associated with significant toxicities, including myelosuppression, cardiotoxicity and nephrotoxicity [11]. Furthermore, the development of drug resistance remains a major obstacle in improving outcomes for patients with recurrent or metastatic disease [12]. These challenges underscore the urgent need for novel therapeutic strategies that can enhance efficacy while minimizing toxicity.

In recent years, biomaterials have emerged as a promising avenue for improving cancer therapy, including the treatment of osteosarcoma [13]. Biomaterials are synthetic or naturally derived substances designed to interact with biological systems for therapeutic or diagnostic purposes [14, 15]. Their unique properties, including biocompatibility, biodegradability and the ability to be functionalized for specific applications, make them ideal candidates for developing advanced drug delivery systems and tissue engineering scaffolds [16, 17].

The application of biomaterials in osteosarcoma treatment offers several potential advantages [18]. First, they can serve as carriers for targeted drug delivery, potentially increasing the concentration of therapeutic agents at the tumor site while reducing systemic toxicity [19]. Second, biomaterials can be engineered to provide controlled and sustained release of drugs, potentially overcoming issues of drug resistance and improving treatment efficacy [20]. Third, in the context of bone tumors, biomaterials can be designed to mimic the extracellular matrix, potentially aiding in bone regeneration following tumor resection [21].

Moreover, the versatility of biomaterials allows for the development of multifunctional platforms that can combine therapeutic delivery with diagnostic capabilities, paving the way for personalized treatment approaches [22]. As research in this field progresses, biomaterials are increasingly being explored for their potential to enhance immunotherapy, facilitate gene therapy and improve the efficacy of conventional chemotherapeutic agents in the treatment of osteosarcoma [20, 23].

This review aims to provide a comprehensive yet focused synthesis of recent progress in biomaterials specifically developed for osteosarcoma treatment. Rather than presenting a general survey of materials for orthopedic or bone repair, we highlight emerging biomaterial strategies uniquely tailored to the complex pathophysiology of osteosarcoma, including multifunctional platforms that integrate antitumor efficacy with bone regeneration, tumor-responsive release systems and immune-modulatory delivery technologies.

We examine advances across multiple therapeutic dimensions, including drug delivery, gene therapy, immunotherapy and post-resection regenerative scaffolds, offering an integrative framework for future translational development. While this review does not aim to be exhaustive, it focuses on representative studies published since 2021, identified through targeted searches in PubMed using terms such as “osteosarcoma,” “biomaterials,” and “bone regeneration.” Priority was given to novel or multifunctional strategies relevant to both tumor inhibition and bone tissue engineering. Reference lists of relevant articles were also consulted to capture key developments.

## Types of biomaterials used in osteosarcoma treatment

**Figure 1:**
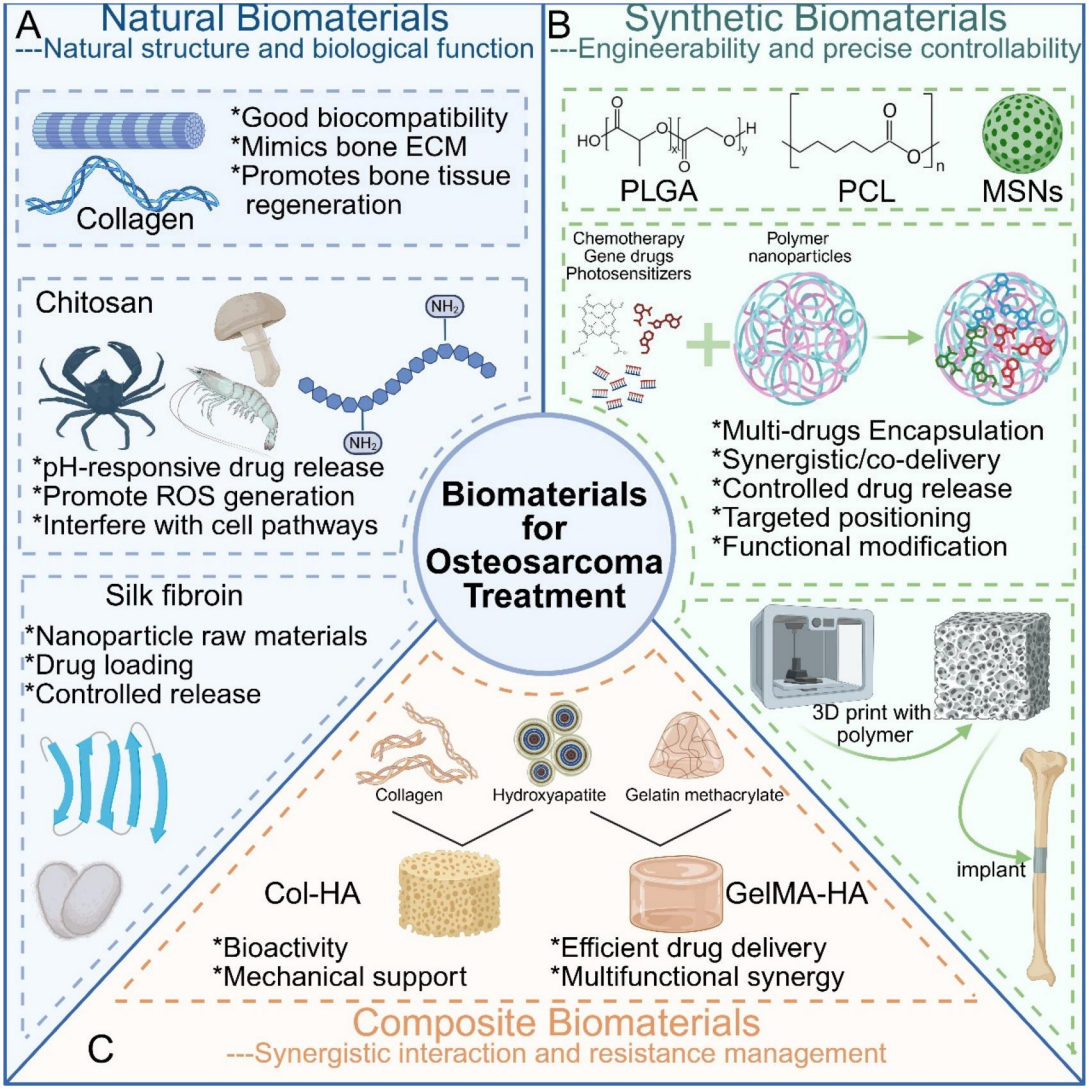
Schematic overview of key biomaterial categories for osteosarcoma treatment. The figure categorizes biomaterials into three major types: natural, synthetic and composite and illustrates their structural characteristics, functional properties and biomedical applications. (**A**) Natural biomaterials (left), including collagen, chitosan and silk fibroin, exhibit excellent biocompatibility and bioactivity, mimicking the ECM of bone while enabling controlled drug release and tumor microenvironment modulation through pH responsiveness and ROS generation. (**B**) Synthetic biomaterials (right), such as PLGA, PCL and MSNs, offer precise engineering flexibility, enabling multidrug encapsulation, targeted delivery and spatiotemporal release through polymer degradation or surface modification strategies. (**C**) Composite biomaterials (bottom), including Col-HA and GelMA-HA, combine the biological functionality of natural polymers with the mechanical tunability and delivery performance of synthetic scaffolds. These systems are often used in 3D printing and post-resection bone implantation. These platforms represent a multifunctional biomaterial toolkit for osteosarcoma management, integrating antitumor delivery with regenerative capabilities. Created with BioRender.com.

The field of biomaterials for osteosarcoma treatment has witnessed significant advancements in recent years, moving beyond traditional materials to encompass a diverse array of innovative solutions. These biomaterials play crucial roles in addressing the complex challenges associated with osteosarcoma, including tumor resection, bone reconstruction and targeted drug delivery. This comprehensive review explores the three main categories of biomaterials used in osteosarcoma treatment: natural biomaterials, synthetic biomaterials and innovative composite biomaterials (Figure 1).

### Natural biomaterials: harnessing bioactivity

Natural biomaterials have gained considerable attention in osteosarcoma treatment due to their inherent bioactivity, biocompatibility and ability to mimic the extracellular matrix (ECM) of bone tissue [13, 24, 25]. These materials offer significant advantages in terms of reducing immune response and enhancing integration with the surrounding bone tissue [26, 27].

Collagen, a primary component of the bone ECM, has been extensively studied for its potential in osteosarcoma treatment [28, 29]. Recent research has demonstrated its efficacy in promoting bone cell proliferation and improving the overall biocompatibility of scaffolds [30, 31]. Studies demonstrated that collagen-I coating of titanium and steel implants via cold low-pressure gas plasma treatment improved biocompatibility [32, 33]. This technique resulted in increased cell viability and attachment rates of osteoblast-like osteosarcoma cells, which could have implications for improving implant integration in osteosarcoma patients. The natural structure of collagen fibers provides an ideal environment for osteoblast attachment and growth, facilitating the regeneration of healthy bone tissue [34, 35]. Gelatin, a denatured form of collagen, has also shown promise in osteosarcoma treatment strategies [36].

Silk fibroin has emerged as a promising material for drug delivery systems in osteosarcoma treatment due to its exceptional properties and versatility [37]. Silk fibroin-based delivery systems have demonstrated remarkable efficacy in targeting and killing tumor cells while minimizing side effects and drug resistance, a crucial factor in improving cancer treatment outcomes [38, 39]. One of the key advantages of silk fibroin is its ability to provide sustained and controlled release of anticancer drugs, which is particularly beneficial for the long-term treatment of osteosarcoma [40]. This material’s versatility allows for the delivery of a wide range of therapeutic molecules, including chemotherapeutics, nucleic acid-based therapies, natural-derived agents, therapeutic proteins or peptides, inorganic compounds and photosensitive molecules [41–43]. Silk fibroin can be formulated into nanoparticles, enhancing drug efficacy through modifications in particle size, chemical composition and properties [44]. Furthermore, these nanoparticles can be functionalized with targeting ligands to improve their specificity for cancer cells, as demonstrated in studies using cyclic RGD peptides to target specific integrin receptors overexpressed in certain cancer types [38, 45, 46].

Chitosan, a versatile biopolymer, has emerged as a promising material in osteosarcoma treatment research, particularly in the development of advanced drug delivery systems [47]. Its ability to form nanoparticles has been extensively studied for encapsulating many therapeutic agents, including chemotherapy drugs and siRNA, offering controlled and sustained release profiles [48]. Research has demonstrated the efficacy of chitosan nanoparticles in delivering siRNA to silence cancer-associated proteins and induce apoptosis in osteosarcoma cells [49, 50]. Additionally, copper-loaded chitosan nanoparticles have shown enhanced anticancer effects through improved cellular internalization and increased reactive oxygen species (ROS) generation [51, 52]. The mechanisms underlying chitosan’s efficacy include enhanced cellular uptake, ROS generation and apoptosis induction in cancer cells [53, 54]. Additionally, chitosan nanoparticles have been found to upregulate ROS generation in bone tumor cells, resulting in mitochondrial dysfunction due to reduced membrane potential [55]. This mitochondrial disruption is a key factor in triggering the apoptotic cascade. Moreover, the ability of chitosan nanoparticles to deliver siRNA effectively allows for targeted silencing of cancer-associated proteins, potentially disrupting multiple oncogenic pathways simultaneously [56]. One notable feature of chitosan-based systems is their pH-responsive nature, allowing accelerated drug release in the acidic tumor microenvironment [57]. The pH-responsive nature of chitosan-based systems not only facilitates drug release in the acidic tumor microenvironment but may also contribute to the disruption of tumor cell homeostasis, further enhancing their therapeutic efficacy [58, 59].

A key insight into the use of natural biomaterials in osteosarcoma treatment is their role in reducing immune response and enhancing integration with bone tissue. The structural similarity of these materials to the native ECM helps minimize foreign body reactions, promoting better acceptance of implants and scaffolds by the host tissue. This improved integration is crucial for successful bone regeneration and long-term treatment outcomes in osteosarcoma patients.

### Synthetic biomaterials: engineering precision for targeted treatment

Synthetic biomaterials offer unparalleled control over material properties, allowing for precise engineering of scaffolds and drug delivery systems tailored to the specific needs of osteosarcoma treatment [60, 61]. These materials provide a platform for customized release profiles of therapeutic agents and can be designed to offer optimal mechanical support for bone regeneration.

Poly (lactic-co-glycolic acid) (PLGA) has emerged as a versatile and promising biomaterial in osteosarcoma treatment, offering significant advantages in drug delivery and scaffold development [62, 63]. Its biocompatibility, biodegradability and ability to form nanoparticles make it an ideal candidate for developing advanced therapeutic strategies [64]. In nanoparticle-based systems, PLGA can encapsulate a wide range of therapeutic agents, including chemotherapy drugs and small molecules, and can be designed for co-delivery of multiple drugs to enhance therapeutic efficacy [65]. These nanoparticles can be further functionalized with targeting ligands to improve tumor-specific delivery [66]. For targeted photodynamic therapy, PLGA nanoparticles can encapsulate photosensitizers and incorporate targeting moieties, allowing for selective accumulation in tumor cells and more effective localized therapy upon light activation [67, 68]. In postsurgical treatment, PLGA-based 3D printed scaffolds provide structural support for bone regeneration while serving as a platform for local drug delivery [23]. The mechanisms of action of PLGA-based systems are multifaceted, including enhanced drug efficacy through improved bioavailability and cellular uptake, increased ROS generation in photodynamic therapy applications, mitochondrial targeting to disrupt cellular energy production, ferroptosis induction and sustained local drug release [69, 70]. These diverse mechanisms make PLGA-based systems highly versatile and effective in osteosarcoma treatment, offering the potential for improved therapeutic outcomes and reduced side effects compared to conventional approaches [71]. The mechanisms of action of PLGA-based systems include enhanced drug efficacy, increased ROS generation, mitochondrial targeting, ferroptosis induction and sustained local drug release. These properties allow PLGA to improve the delivery and efficacy of chemotherapeutic agents, overcome drug resistance mechanisms and maintain therapeutic concentrations at the tumor site while minimizing systemic side effects.

Polycaprolactone (PCL) is another synthetic polymer that is a biodegradable polyester with excellent biocompatibility and mechanical properties, making it an ideal candidate for developing advanced therapeutic strategies in osteosarcoma treatment [72]. Its primary functions include serving as a drug delivery vehicle and a scaffold material for tissue engineering applications [73]. In drug delivery applications, PCL can be formulated into nanoparticles or microparticles to encapsulate various therapeutic agents [74]. These PCL-based delivery systems offer controlled and sustained release of drugs, potentially improving the efficacy of chemotherapy while reducing systemic side effects [75]. For instance, a study explored the use of PCL nanoparticles for delivering peptides, demonstrating the potential for enhanced drug biodistribution in targeted therapies [76]. As a scaffold material, PCL plays a crucial role in tissue engineering approaches for bone regeneration following osteosarcoma resection [77]. PCL scaffolds provide a structural framework that supports cell growth and differentiation while allowing for the controlled release of therapeutic agents. A notable example is the development of 3D-printed magnesium-PCL scaffolds loaded with melatonin for osteosarcoma treatment [78]. This innovative approach not only provided structural support but also actively inhibited osteosarcoma development by regulating cell-in-cell structures. An additional benefit of PCL in osteosarcoma treatment is its potential for antibacterial properties when combined with other materials. A study demonstrated enhanced antibacterial ability of electro spun PCL scaffolds incorporating ZnO nanowires [79]. This property could be particularly valuable in preventing postsurgical infections in osteosarcoma patients undergoing limb-salvage procedures.

Mesoporous silica nanoparticles (MSNs) represent a class of synthetic biomaterials that have shown great potential in improving the stability and delivery of therapeutic agents for osteosarcoma treatment [80]. Their high surface area and tunable pore size allow for efficient loading of drugs and controlled release profiles [81]. Recent research has demonstrated the efficacy of MSNs in delivering photosensitizers like indocyanine green (ICG) for photothermal therapy in osteosarcoma treatment, showcasing their versatility in advanced treatment modalities [82]. MSNs can be functionalized with targeting ligands to improve their uptake by osteosarcoma cells [83]. MSNs can be designed to overcome drug resistance mechanisms in osteosarcoma cells by co-delivering chemosensitizers or targeting specific resistance pathways. A study developed MSNs loaded with DOX and conjugated with a selenium to overcome multidrug resistance in osteosarcoma cells [84]. MSNs can be engineered to alter the tumor microenvironment, potentially enhancing the efficacy of other treatments. This approach enhanced the efficacy of subsequent radiotherapy. Unlike many materials that can only function therapeutically, MSNs can also be functionalized for diagnostic applications in osteosarcoma [85]. A study created multifunctional MSNs loaded with DOX and gadolinium for simultaneous MRI imaging and chemotherapy of osteosarcoma [85]. This system demonstrated enhanced tumor accumulation and antitumor efficacy while providing real-time imaging capabilities.

A standout feature of synthetic biomaterials in osteosarcoma treatment is their tunability for controlled drug release and bone support. The ability to precisely engineer the physical and chemical properties of these materials allows for the development of sophisticated drug delivery systems with customized release profiles. This level of control is particularly valuable in osteosarcoma treatment, where maintaining therapeutic drug concentrations at the tumor site while minimizing systemic exposure is crucial.

### Innovative composite biomaterials: synergizing for enhanced functionality

In osteosarcoma treatment, the integration of natural biomaterials and synthetic biomaterials into innovative composite biomaterials has emerged as a promising approach, offering significant advantages over their individual counterparts [21]. These composite materials exhibit enhanced biocompatibility, improved mechanical properties and superior drug delivery capabilities, addressing the limitations of single-component systems [86]. By combining the bioactivity and cell-friendly nature of natural biomaterials with the tailorable properties and stability of synthetic biomaterials, these innovative composites provide a versatile platform for targeted drug delivery, sustained release of therapeutic agents and promotion of bone regeneration.

Collagen-hydroxyapatite (COLL-HA) composites are designed to mimic the natural composition of bone, typically consisting of collagen and hydroxyapatite (HAp) in ratios similar to that of bone tissue [87]. The incorporation of collagen provides a biocompatible matrix, while HAp contributes to the mechanical strength and osteoconductive of the composite. COLL-HA composites have been investigated as drug delivery systems for the locoregional treatment of bone cancer, particularly osteosarcoma. Researchers have developed COLL-HA composites loaded with cisplatin, a common chemotherapeutic agent [88, 89]. These systems demonstrated cytotoxic, antiproliferative and anti-invasive activities against G292 osteosarcoma cells *in vitro*. Another research developed a porous COLL-HA scaffolds containing drug-loaded Adriamycin (ADM)-encapsulated PLGA nanoparticles have been explored for osteosarcoma treatment, and exhibited excellent extended-release drug properties, bone repairing and antineoplastic efficacy [90].

In addition to its common combination with collagen, HAp can be blended with a variety of other bioactive substances to create innovative and multifunctional composite biomaterials for osteosarcoma treatment [91]. These diverse composites not only enhance the mechanical and biological properties of the materials but also introduce unique therapeutic benefits. One innovative approach involves selenium-loaded HAp nanoparticles, which have shown both anticancer and osteogenic properties by effectively inhibiting the activity and invasiveness of osteosarcoma cells while promoting the viability and mineralization of bone marrow stem cells [92]. *In vivo* studies demonstrated a significant reduction in tumor growth in mouse models, indicating their potential as a direct treatment option that activates apoptotic pathways in tumor cells. Another promising development is a bioinspired hydrogel enriched with calcium ions that forms a dense HAp layer, selectively targeting and killing osteosarcoma cells while sparing normal cells, thereby addressing issues like multidrug resistance associated with conventional chemotherapy [93, 94]. Additionally, HAp combined with bovine serum albumin and paclitaxel has been formulated into nanoparticles aimed at postsurgical treatment, inhibiting osteosarcoma cell proliferation and invasion while promoting osteogenic differentiation, making them suitable for adjuvant therapy after surgery [95]. Furthermore, bismuth/strontium/HAp/chitosan composites have been developed to exhibit antitumor, antibacterial and osteogenic properties, enhancing effectiveness against osteosarcoma cells while also providing antibacterial benefits to mitigate infection risks following surgical interventions [96]. Overall, HAp-containing composites represent a promising area of research in the direct treatment of osteosarcoma, demonstrating capabilities that not only support bone regeneration but also actively inhibit tumor growth and enhance healing processes postsurgery. As research advances, these innovative composites may lead to improved therapeutic strategies for managing osteosarcoma effectively.

Gelatin methacryloyl (GelMA) is a versatile and biocompatible hydrogel derived from gelatin that has gained significant attention in tissue engineering and regenerative medicine [97]. It is created by modifying gelatin with methacrylic anhydride, introducing methacrylate groups that allow for photo-crosslinking [98]. GelMA hydrogels offer tunable mechanical properties, excellent biocompatibility and the ability to promote cell adhesion due to retained RGD motifs [99, 100]. GelMA hydrogels are very special in the treatment of osteosarcoma, because in addition to their wide range of direct use as treatment, GelMA hydrogels occupy a very key position in the research stage of osteosarcoma treatment, providing a valuable tool for understanding the disease and developing new treatment strategies. GelMA hydrogels enable the creation of more physiologically relevant 3D osteosarcoma models [101]. These models enable more accurate drug screening, facilitate studies on metastasis and mechanobiology and support the development of personalized medicine approaches. GelMA-based 3D cultures allow researchers to study complex cell-cell interactions, tumor heterogeneity and cell–matrix interactions in a more realistic microenvironment [102, 103]. They serve as effective platforms for high-throughput drug screening, metastasis studies and investigation of mechanotransduction in osteosarcoma cells. Additionally, GelMA hydrogels support the creation of patient-specific models and the study of combination therapies, including multidrug delivery and chemo-radiotherapy models. As research techniques continue to evolve, GelMA-based models are likely to become increasingly important in uncovering new insights into osteosarcoma biology and developing more effective treatment strategies.

With these capabilities, composite biomaterials allow the development of multifunctional treatment modalities that not only suppress tumor growth and facilitate bone repair but also help minimize systemic side effects [104]. Their synergistic design makes them well-suited for personalized, localized and sustained therapies, reinforcing their promise in transforming current osteosarcoma treatment paradigms. Overall, the field of biomaterials for osteosarcoma treatment has evolved significantly, embracing a diverse array of natural, synthetic and composite materials. Natural biomaterials offer excellent biocompatibility and bioactivity, synthetic materials provide precision and tunability, and composite biomaterials synergize these advantages for enhanced functionality. As research in this field continues to advance, the development of increasingly sophisticated biomaterials promises to revolutionize osteosarcoma treatment, offering new hope for improved patient outcomes and quality of life (Table 1).

**Table 1:** Comparative summary of representative biomaterial platforms for osteosarcoma treatment.

Biomaterial	Physicochemical properties	Typical therapeutic payloads	*In vivo* efficacy
**Collagen**	Biocompatible; ECM mimic; low mechanical strength	Cisplatin, DOX	Promotes osteoblast adhesion; supports osteogenesis
**Gelatin**	Derived from collagen; biodegradable; cell-adhesive	Various chemotherapeutics	Enhances scaffold biocompatibility; supports tissue integration
**Silk Fibroin**	Biodegradable; tunable drug release; nanoparticle-compatible	Chemotherapeutics, siRNA, peptides	Sustained release; reduced resistance; improved targeting
**Chitosan**	Cationic; pH-responsive; forms nanoparticles	siRNA, DOX, Cu^2+^	Induces ROS; triggers apoptosis; gene delivery capability
**PLGA**	Biodegradable; tunable degradation rate; nanoparticle-friendly	DOX, methotrexate, photosensitizers	Controlled local drug release; improved bioavailability
**PCL**	Biodegradable polyester; strong mechanical properties; 3D printable	Melatonin, peptides	Supports bone regeneration; antibacterial when modified
**MSNs**	High surface area; tunable pores; surface-functionalizability	DOX, selenium, ICG	Combines therapy and imaging; overcomes drug resistance
**COLL-HA**	Bone-mimetic; osteoconductive composite	Cisplatin, DOX-PLGA NPs	Promotes bone regeneration and tumor suppression
**GelMA**	Photo-crosslinkable hydrogel; tunable stiffness; RGD motifs	PEDF gene, multiple antitumor drugs	Enables 3D tumor modeling; sustained gene and drug delivery

## Biomaterial-based delivery systems for osteosarcoma treatment

**Figure 2:**
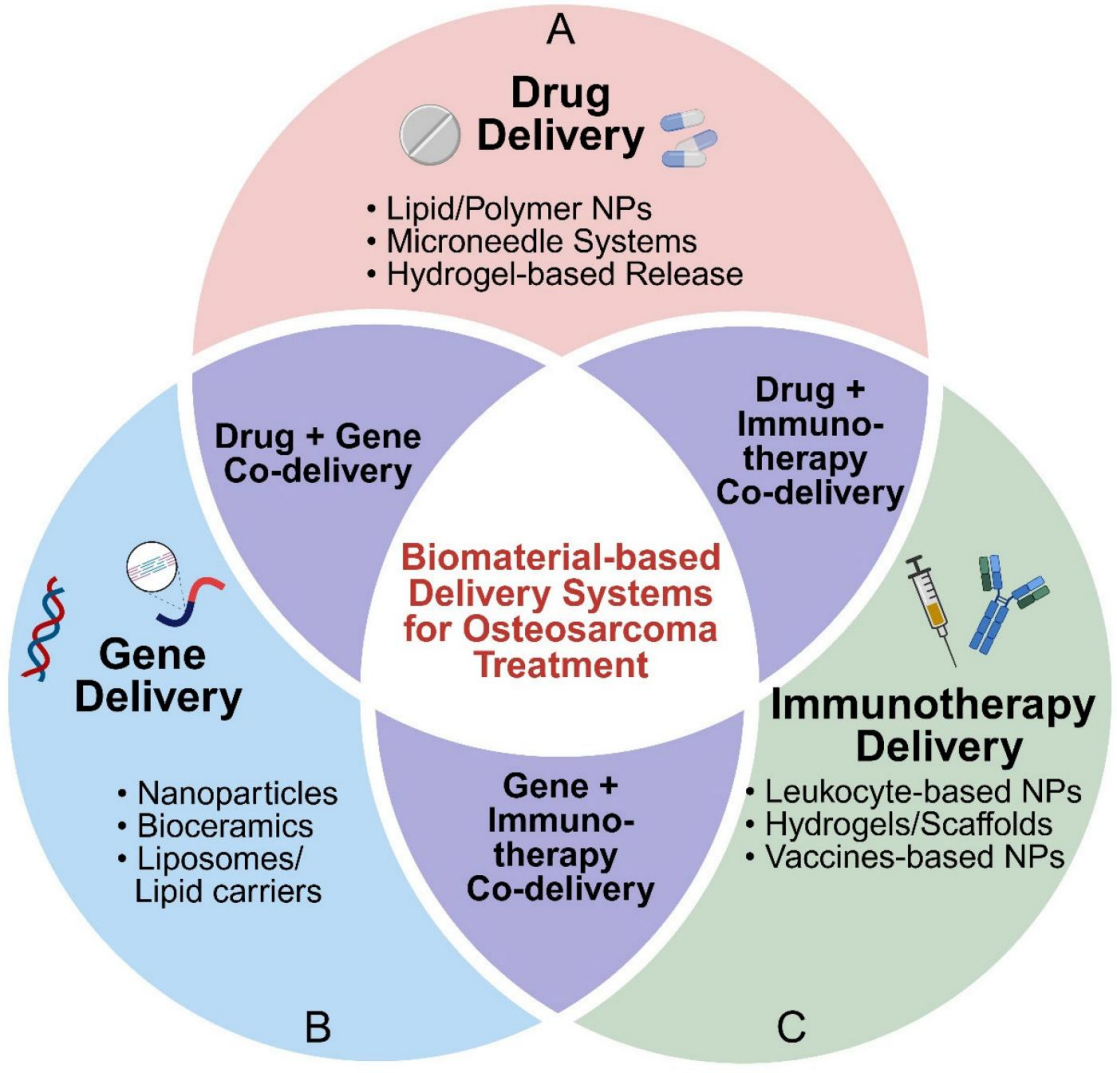
Schematic overview of biomaterial-based delivery systems for osteosarcoma treatment. Biomaterial-based delivery platforms can be broadly categorized into drug delivery, gene delivery and immunotherapy/vaccine delivery systems, with increasing interest in multifunctional co-delivery strategies that occupy the intersection of these domains. (**A**) Drug delivery systems utilize lipid- or polymer-based nanoparticles, microneedles and hydrogel matrices to enable controlled and localized release of chemotherapeutic agents. (**B**) Gene delivery systems employ vectors such as nanoparticles, liposomes or bioceramics to deliver DNA, siRNA or miRNA for tumor suppression or gene silencing. (**C**) Immunotherapy delivery platforms include leukocyte-mimetic nanoparticles, injectable hydrogels and vaccine-loaded carriers to activate antitumor immune responses through checkpoint modulation or antigen presentation. Co-delivery strategies (center) leverage combinatorial effects, for instance, synchronizing chemotherapy with gene silencing or immune activation, to overcome drug resistance and enhance therapeutic efficacy. These integrated platforms reflect a growing trend toward synergistic biomaterial systems capable of addressing multiple therapeutic targets in osteosarcoma. Created with BioRender.com.

Despite advances in treatment modalities, the high metastatic potential, recurrence rates and development of drug resistance in osteosarcoma continue to pose significant challenges. To address these issues, biomaterial-based delivery systems have emerged as promising approaches for enhancing therapeutic efficacy while minimizing adverse effects in osteosarcoma treatment. These systems can be broadly categorized into drug delivery systems, gene delivery systems and immunotherapy and vaccination delivery systems (Figure 2).

### Drug delivery systems

**Figure 3:**
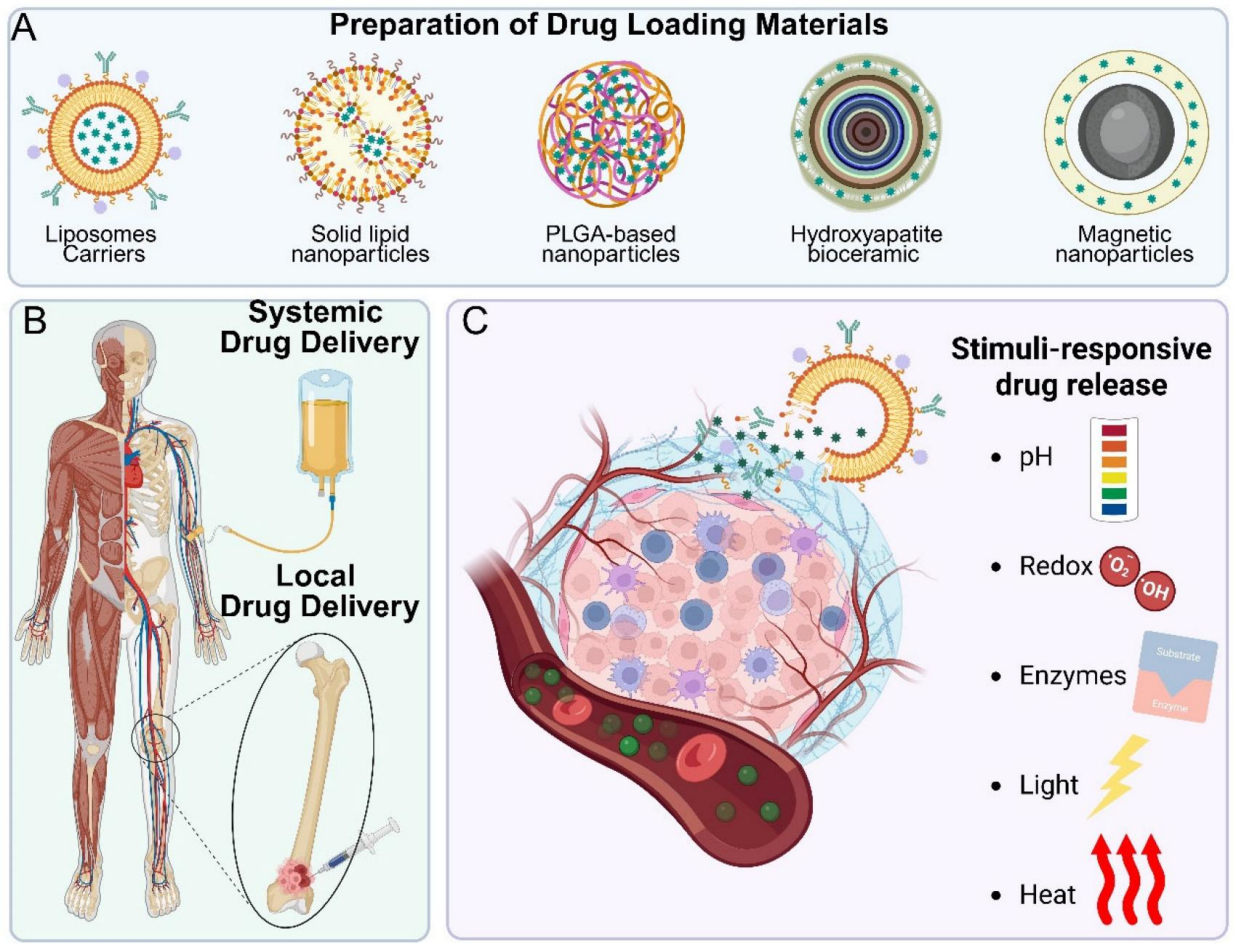
Overview of advanced biomaterial-based drug delivery strategies for osteosarcoma. This figure illustrates the key components and mechanisms involved in the design and function of biomaterial-assisted drug delivery systems. (**A**) Various nanomaterials and bioceramics carriers, including liposomes, solid lipid nanoparticles, PLGA-based nanoparticles, hydroxyapatite bioceramics and magnetic nanoparticles, are employed as delivery platforms to encapsulate and protect therapeutic agents while enabling tunable release profiles. (**B**) Two administration routes are shown: systemic delivery (e.g. intravenous infusion), which allows broad distribution but often results in off-target effects, and localized delivery, which involves direct injection at the tumor site, enabling higher local concentrations and reduced systemic toxicity. (**C**) Stimuli-responsive drug release strategies are increasingly used to improve therapeutic specificity. These include materials engineered to respond to tumor-associated triggers such as acidic pH, oxidative stress, overexpressed enzymes or exogenous cues like light and heat. Upon sensing these stimuli, carrier systems undergo structural changes (e.g. membrane destabilization, matrix degradation), facilitating site-specific drug release within the tumor microenvironment. Created with BioRender.com.

Drug delivery systems for osteosarcoma treatment have seen significant advancements in recent years, leveraging various biomaterials to improve the efficacy and safety of chemotherapeutic agents [105]. These systems aim to overcome limitations associated with conventional drug administration, such as poor bioavailability, rapid clearance and off-target toxicity (Figure 3) [47].

Calcium phosphate-based bioceramics, particularly HAp composites, have demonstrated potential as carriers for antitumor drugs like DOX, cisplatin and gemcitabine [106]. The similarity of HAp to natural bone mineral makes it an excellent candidate for targeted delivery to bone tumors [13]. Moreover, HAp scaffolds combined with magnetic nanoparticles (MNPs) offer the possibility of local magnetic hyperthermia treatment, enhancing the overall therapeutic effect [107]. This combination approach allows for both drug delivery and thermal ablation of tumor cells, potentially improving treatment outcomes. Biodegradable polymers, especially those based on lactic acid, have shown potential as alternatives to metallic implants in bone implant technology, offering a dual function of drug delivery and structural support [108]. PLGA and PCL are commonly used polymers in this context. These polymeric systems can be engineered to provide controlled release of chemotherapeutic agents, potentially overcoming drug resistance mechanisms and reducing systemic toxicity [23]. The degradation rate of these polymers can be tuned to match the desired drug release profile and bone regeneration timeline. Lipid-based nanoparticles and liposomal formulations have been extensively explored for their ability to improve drug solubility and enhance targeted delivery to osteosarcoma sites [109]. Solid lipid nanoparticles (SLNs) and nanostructured lipid carriers (NLCs) offer advantages such as high drug loading capacity, improved stability and the ability to incorporate both hydrophilic and hydrophobic drugs [110]. Liposomal formulations, particularly those incorporating polyethylene glycol (PEG) for “stealth” properties, have shown improved pharmacokinetics and reduced systemic toxicity in osteosarcoma models [23, 109].

The development of stimuli-responsive drug delivery systems has further expanded the possibilities for controlled and targeted drug release in osteosarcoma treatment [111]. pH-sensitive nanoparticles and hydrogels have been designed to trigger drug release in the acidic tumor microenvironment, potentially enhancing therapeutic efficacy while minimizing off-target effects [112]. This approach takes advantage of the lower pH typically found in tumor tissues compared to normal tissues. Thermosensitive systems, such as certain polymeric nanoparticles and liposomes, can be triggered to release their payload in response to local hyperthermia, allowing for precise spatial and temporal control of drug delivery [113]. This strategy can be particularly useful when combined with other treatment modalities like radiation therapy or focused ultrasound [114]. Recent advancements in nanotechnology have led to the development of multifunctional nanoparticles that combine diagnostic and therapeutic capabilities, known as theranostic nanoparticles. For instance, iron oxide nanoparticles functionalized with chemotherapeutic drugs can serve as both MRI contrast agents and drug delivery vehicles, enabling real-time monitoring of drug distribution and tumor response [85].

In brief, recent advancements in drug delivery systems for osteosarcoma treatment utilize biomaterials, polymers and nanotechnology to improve targeted delivery and controlled drug release. These innovations aim to enhance therapeutic efficacy, minimize toxicity and integrate diagnostic and therapeutic capabilities.

### Gene delivery systems

Gene therapy holds significant promise for osteosarcoma treatment, offering the potential to target specific genetic alterations associated with tumor growth and progression [115]. However, effective delivery of genetic material to tumor cells remains a critical challenge. Biomaterial-based gene delivery systems have been developed to address this issue, providing protection for nucleic acids and facilitating their cellular uptake and intracellular release [116].

**Figure 4:**
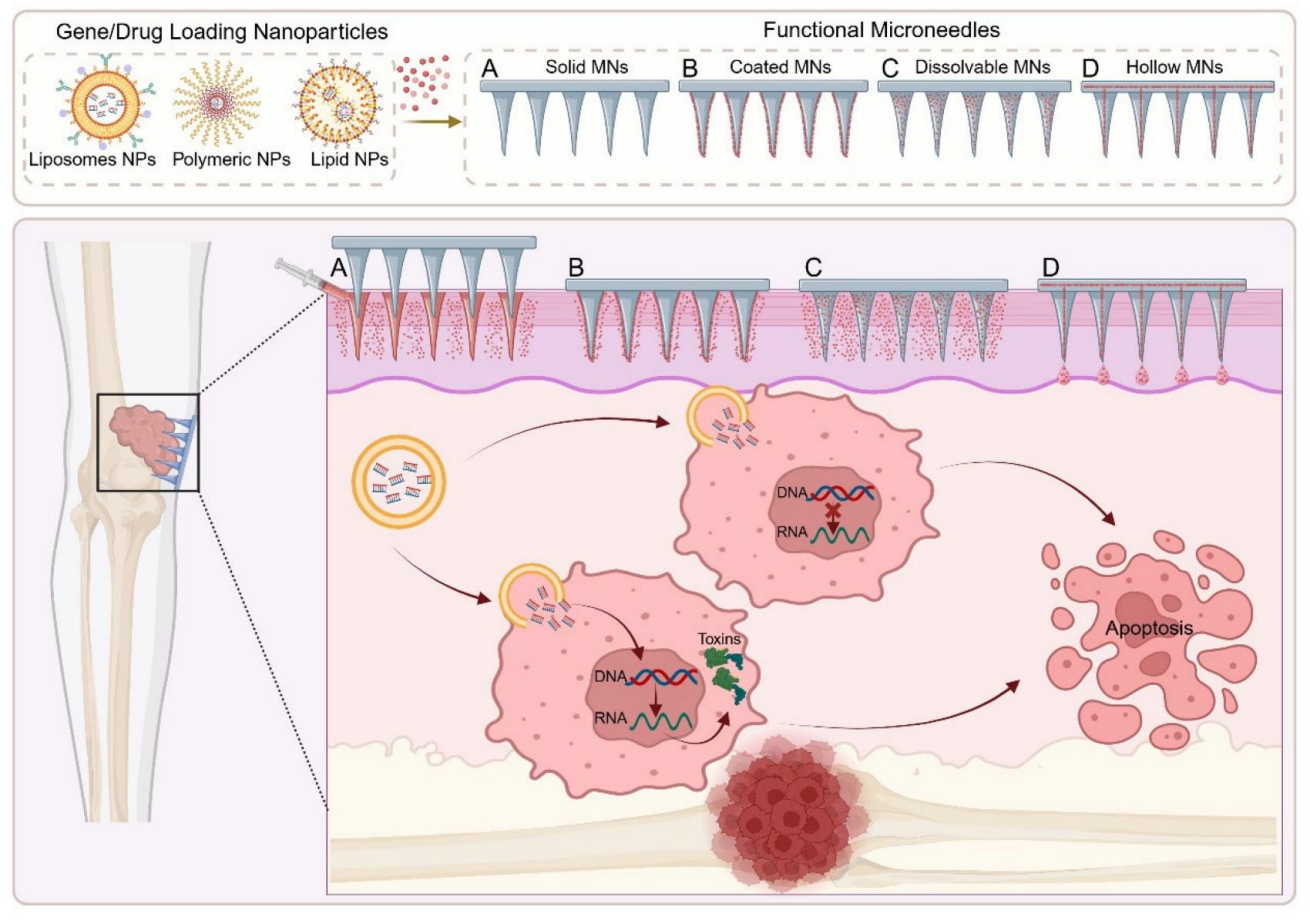
Schematic illustration of microneedle‐based gene therapy for osteosarcoma. This figure depicts the localized delivery of gene- or drug-loaded nanoparticles using MN platforms for targeted osteosarcoma treatment. The nanoparticles encapsulated within liposomes, polymeric or lipid-based carriers are incorporated into four functional microneedle designs: (**A**) Solid MNs create microchannels in the skin to facilitate passive nanoparticle diffusion; (**B**) Coated MNs deliver therapeutic agents via surface-loaded cargo that dissolves rapidly upon insertion; (**C**) Dissolvable MNs are fully biodegradable, releasing their entire payload as the needle matrix dissolves; (**D**) Hollow MNs actively inject nanoparticle suspensions through internal lumens. Once delivered, the nanoparticles penetrate tumor cells, enabling gene regulation (e.g. silencing oncogenes or delivering tumor-suppressor genes) and promoting apoptosis through transcriptional disruption or toxin induction. This approach enables minimally invasive, localized and efficient nucleic acid delivery directly to bone tumor sites, reducing systemic toxicity and improving therapeutic precision. Created with BioRender.com.

Microneedle-guided delivery systems have emerged as an innovative approach for gene therapy in osteosarcoma (Figure 4) [117]. Dissolving microneedles (dMN) based on biocompatible amphiphilic tri-block copolymers enable the self-assembly of nano-micelles containing hydrophobic drugs or genetic material [118]. This system has shown potential for delivering gene-based therapies to skin tumors and osteosarcoma, offering a minimally invasive approach for localized gene delivery. Targeted drug delivery for osteosarcoma treatment, showing nanoparticles entering tumor cells, releasing chemotherapeutic agents, damaging DNA and RNA and ultimately inducing apoptosis to eliminate the osteosarcoma cells [119].

Hydrogel-based systems have also demonstrated promise for gene delivery in osteosarcoma treatment [120]. A notable example of hydrogel use in gene therapy for osteosarcoma treatment involves a chitosan-based hydrogel system designed to deliver the pigment epithelium-derived factor (PEDF) gene [121]. This *in situ* gelling system forms at the application site, allowing for localized and sustained release of the therapeutic gene. The hydrogel’s key features include controlled release of the PEDF gene, targeted delivery to the tumor site, potential for combination with conventional chemotherapy, and minimally invasive application. This approach demonstrates how hydrogels can serve as versatile platforms for gene therapy in osteosarcoma treatment, offering precise delivery and the potential for enhanced therapeutic efficacy.

Nanoparticle-based systems, including lipid and polymer-based nanocarriers, have been extensively studied for gene delivery in osteosarcoma. These systems can protect nucleic acids from degradation, facilitate cellular uptake and enable targeted delivery to tumor sites [122]. Cationic lipid nanoparticles, for example, have shown efficacy in delivering siRNA and miRNA for gene silencing in osteosarcoma models [123, 124]. Polymer-based nanocarriers, such as those based on polyethyleneimine (PEI) or poly(amidoamine) (PAMAM) dendrimers, have also demonstrated potential for efficient gene transfection in osteosarcoma cells [13].

Gene therapy for osteosarcoma offers promising strategies to target tumor-specific genetic alterations, with advancements in biomaterial-based delivery systems like microneedles, hydrogels and nanoparticles enabling effective and localized gene delivery. These systems enhance therapeutic efficacy by protecting genetic material, facilitating targeted delivery and offering minimally invasive application methods.

### Immunotherapy and vaccination delivery systems

Immunotherapy has emerged as a promising approach for osteosarcoma treatment, aiming to harness the power of the immune system to combat cancer cells. Biomaterial-based delivery systems play a crucial role in enhancing the efficacy of immunotherapeutic agents and vaccines by providing controlled release, protection from degradation and targeted delivery to immune cells [61].

Leukocyte-based biomimetic nanoparticles, such as leukosomes, have shown potential for combined targeted and immunotherapy approaches in osteosarcoma [125]. Leukosomes can effectively deliver multi-tyrosine kinase inhibitors (mTKIs) to induce an immunogenic response in osteosarcoma. These nanoparticles can deliver immunomodulatory agents while evading immune clearance, potentially enhancing the overall immune response against tumor cells.

Cholesterol-bearing polysaccharide-based nanogels have demonstrated utility as drug delivery systems and scaffolds for immunotherapy and regenerative medicine [126]. These nanogels can encapsulate and release immunomodulatory agents, providing a versatile platform for enhancing antitumor immune responses. Lipid-based nanoparticle combination immunotherapy systems have also shown promise, combining agonists of the Stimulator of Interferon Genes (STING) and Toll-like Receptor 4 (TLR4) pathways to promote antitumor immunity [127, 128]. Hydrogel-based systems, such as methacrylated glycol chitosan (MGC) hydrogels, have demonstrated the ability to activate tumor-related immune cells both locally and over a prolonged period, enhancing immunotherapeutic efficacy [129]. These hydrogels can serve as depots for the sustained release of immunomodulatory agents, potentially overcoming the limitations of systemic administration.

Biomaterial-based vaccine delivery systems have also been explored for osteosarcoma treatment. These systems can enhance the stability and immunogenicity of tumor-associated antigens, potentially eliciting more robust and durable antitumor immune responses. Nanoparticle-based vaccine formulations, including those based on PLGA or liposomes, have shown promise in delivering tumor antigens and adjuvants to antigen-presenting cells, promoting effective T cell responses against osteosarcoma [130]. Moreover, checkpoint inhibitor delivery platforms, such as hydrogels and microspheres designed to locally release anti-PD-1 or anti-PD-L1 antibodies, have shown promise in enhancing intratumoral immune activation while reducing systemic toxicity. For example, thermosensitive hydrogels loaded with anti-PD-L1 have demonstrated improved tumor infiltration of cytotoxic T cells in murine osteosarcoma models. In addition, biomaterial-assisted CAR-T cell delivery is an emerging strategy aimed at improving cell retention and activation within the tumor microenvironment. While CAR-T therapy for solid tumors like osteosarcoma remains challenging due to poor infiltration and immunosuppression, biodegradable scaffolds and nanoparticles have been investigated as supportive platforms to modulate local cytokine levels and improve T-cell viability.

**Figure 5:**
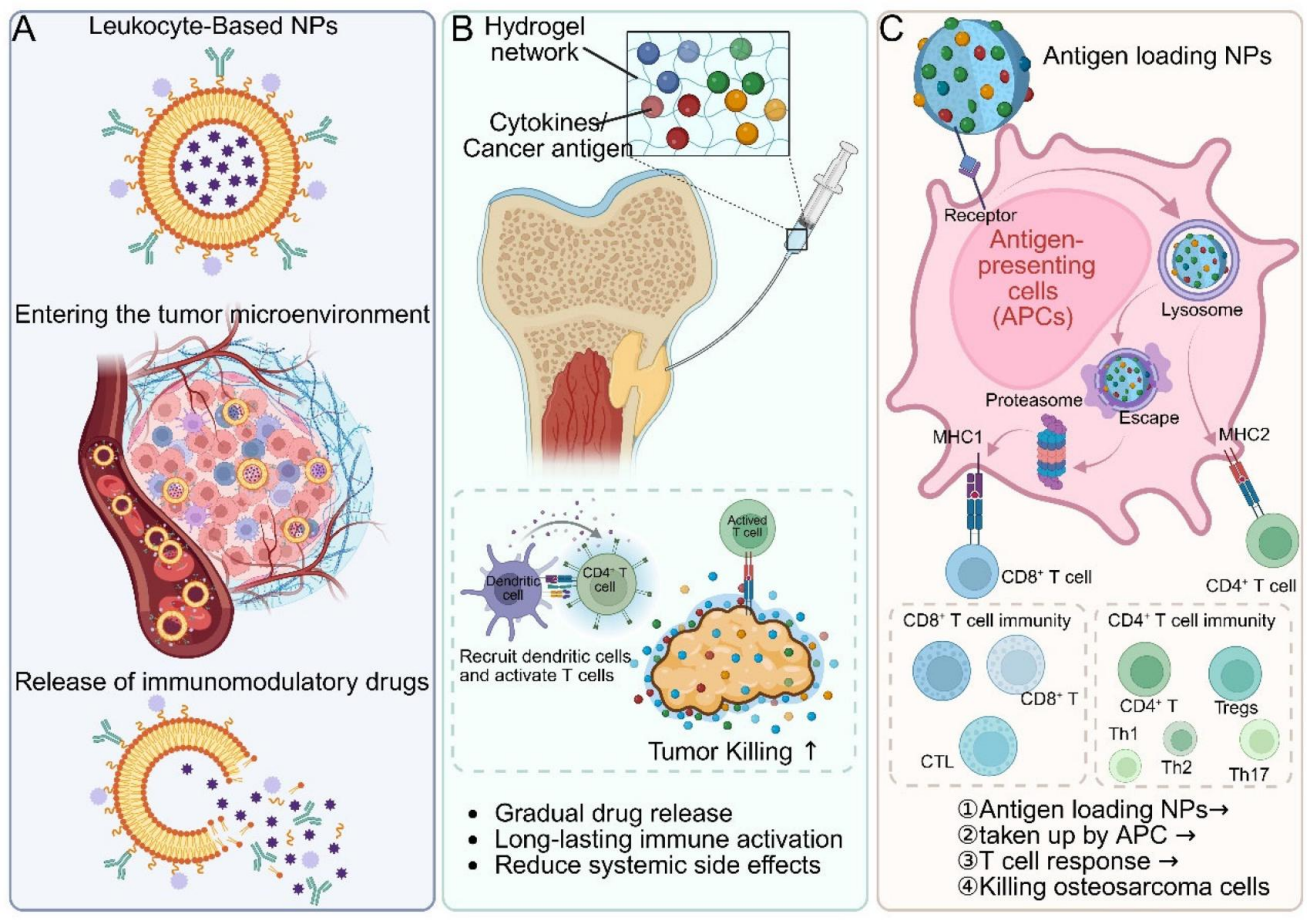
Schematic illustration of biomaterial-based immunotherapy strategies for osteosarcoma. This figure summarizes key delivery platforms and mechanisms by which biomaterials enhance antitumor immune responses in osteosarcoma. (**A**) Leukocyte-mimetic NPs exploit immune evasion and targeting properties to accumulate in the tumor microenvironment, where they gradually release immunomodulatory agents (e.g. checkpoint inhibitors or cytokines) to reshape the local immune milieu. (**B**) Injectable hydrogels act as localized depots for sustained release of cytokines and tumor-associated antigens. These hydrogels recruit dendritic cells, which present antigens and activate CD4^+^ T cells and CTLs, enhancing localized immune activation and minimizing systemic exposure. (**C**) Antigen-loaded nanoparticles are internalized by APCs, escape lysosomal degradation and process antigens through both MHC-I and MHC-II pathways. This dual presentation activates CD8^+^ cytotoxic T cells and CD4^+^ helper T cell subtypes (e.g. Th1, Th17), resulting in a coordinated antitumor response. These biomaterial-enabled immunotherapies offer a promising strategy to enhance tumor-specific immunity, extend therapeutic duration and reduce off-target effects in osteosarcoma treatment. Created with BioRender.com.

Immunotherapy for osteosarcoma leverages biomaterial-based delivery systems, such as nanoparticles, nanogels, hydrogels and vaccines, to enhance immune responses by ensuring targeted delivery, sustained release and protection of immunotherapeutic agents (Figure 5). These innovations aim to improve antitumor immunity and overcome the limitations of conventional systemic therapies.

In conclusion, biomaterial-based delivery systems offer diverse and promising approaches for enhancing the efficacy of drug delivery, gene therapy and immunotherapy in osteosarcoma treatment. These systems leverage the unique properties of various biomaterials to overcome challenges associated with traditional therapies, such as poor bioavailability, off-target effects and limited efficacy. As research in this field continues to advance, the integration of these innovative approaches into clinical practice holds great promise for improving outcomes for osteosarcoma patients. Future directions may include the development of multifunctional delivery systems that combine different therapeutic modalities, as well as personalized approaches tailored to individual patient characteristics and tumor profiles. Continued research and interdisciplinary collaboration will be crucial for translating these promising biomaterial-based strategies into effective clinical treatments for osteosarcoma.

### Smart/stimuli-responsive biomaterials

Innovative biomaterials employed in osteosarcoma therapy often incorporate controlled drug release mechanisms to improve treatment precision and minimize systemic toxicity [131]. These systems typically operate through a combination of passive and responsive modalities. In many polymeric carriers and porous scaffolds, drug release is primarily governed by diffusion and the gradual degradation of the matrix material, as seen in widely used PLGA and PCL systems, allowing for sustained and programmable drug exposure over time [16].

Building upon these strategies, stimuli-responsive or “smart” biomaterials have rapidly emerged as promising therapeutic platforms for osteosarcoma due to their capacity to dynamically respond to specific internal or external cues in the tumor microenvironment [132]. Unlike conventional passive drug delivery systems, smart biomaterials are designed to recognize and respond to pathophysiological signals such as pH gradients, ROS, specific enzymes, temperature shifts or redox conditions [133]. This allows for spatiotemporally controlled drug release, enhanced therapeutic specificity, reduced systemic toxicity and even integration of diagnostic and therapeutic functionalities. The osteosarcoma microenvironment, characterized by acidity, oxidative stress, enzyme overexpression and hypoxia, offers distinct molecular targets that make smart biomaterials particularly advantageous in overcoming challenges such as drug resistance, poor targeting and severe side effects.

Among these systems, pH-responsive platforms are the most extensively studied. For example, Zhu *et al*. developed a pH-sensitive hydrogel based on a self-assembling nonapeptide (P1) for targeted delivery of doxorubicin (DOX). The hydrogel remained stable under physiological pH but significantly accelerated DOX release under acidic conditions (3.6-fold higher release at pH 5.8 than at pH 7.4) [134]. *In vivo* studies demonstrated enhanced DOX accumulation at tumor sites and superior antitumor efficacy, highlighting its therapeutic potential and biocompatibility. Similar pH-responsive systems using chitosan-based nanocarriers have also been applied for siRNA delivery to silence key oncogenes within osteosarcoma cells.

ROS-responsive systems exploit elevated intracellular ROS levels in tumor cells as a trigger for drug release [135]. A representative example is the development of a self-adaptive, multifunctional RPSH hydrogel incorporating dual-responsive R/I@SeNP nanoparticles (composed of RRx-001 and ICG) into a PAAm/SA/HA matrix [136]. This hydrogel effectively modulated the postsurgical inflammatory microenvironment by inducing M2 macrophage polarization and triggered the release of immunomodulatory species such as RSeO(OH) upon ROS stimulation, achieving a tumor inhibition rate of 72.84%. Additionally, long-term application of the hydrogel promoted robust bone regeneration, with a BV/TV ratio reaching 59.03% after eight weeks, underscoring its dual functionality in immunomodulation and osteogenesis.

Thermo-responsive systems have also been investigated, primarily for use in combinatorial photothermal therapy and localized chemotherapy [137]. An injectable thermosensitive hydrogel (mPEG_45_–PLV_19_) was developed to co-deliver methotrexate and alendronate [138]. This hydrogel rapidly solidified at body temperature, provided sustained drug release and demonstrated excellent physicochemical properties. *In vivo* results showed significant inhibition of tumor growth, reduction in bone destruction and suppression of lung metastasis, indicating great potential for osteosarcoma chemotherapy.

Studies have also focused on integrating multiple stimuli-responsive mechanisms within a single system to enable synergistic control and combinatorial therapy. This approach holds promise for enhancing precision and efficacy in complex tumor microenvironments. In addition, smart biomaterials are increasingly applied in osteosarcoma immunotherapy and gene therapy [139]. Hydrogels responsive to inflammatory cues have been designed to locally release immunomodulators, such as STING or TLR4 agonists, to activate tumor-specific immune responses. Redox-sensitive liposomes or polymeric nanoparticles have also been employed to deliver siRNA, effectively silencing oncogenic pathways in osteosarcoma cells and offering more targeted and safer gene therapy strategies [140].

Despite the great potential of smart biomaterials, several challenges hinder their clinical translation. These include complex material design, difficulties in large-scale manufacturing, inter-patient variability in stimulus profiles and concerns regarding the long-term biocompatibility and safety of certain responsive components. Moreover, multifunctional systems often face regulatory ambiguity, complicating their path to approval. Future research should prioritize modular design strategies to improve production reproducibility, simplify response mechanisms and utilize fully degradable, nontoxic materials. Interdisciplinary collaboration between materials science, biomedical engineering and clinical oncology will be critical to establish streamlined pathways from *in vivo* validation to GMP-grade production.

In summary, the integration of smart biomaterials marks a change in thinking in osteosarcoma management, from passive treatment to precision-responsive, multifunctional therapy. By sensitively responding to microenvironmental cues, these systems offer enhanced therapeutic outcomes, combination strategies and reduced toxicity, holding promise as a central component in the future landscape of osteosarcoma treatment alongside drug, gene and immune-based therapies.

To provide a more systematic perspective on recent advances, we compiled a summary of representative studies from 2021 to 2025 that explore biomaterial-based delivery strategies in osteosarcoma therapy. Table 2 presents an overview of 64 original research articles, facilitating comparative analysis across biomaterial types, therapeutic payloads and functional mechanisms. This classification underscores the translational potential of multifunctional delivery systems and helps identify promising directions for future development. Notably, the included studies cover both single-material delivery systems, such as polymeric nanoparticles, liposomes or hydrogels and integrated platforms that combine multiple biomaterial types with synergistic therapeutic modalities. These multifunctional constructs often couple drug delivery with gene or immunotherapy, offering more comprehensive approaches to overcome tumor resistance and enhance localized treatment efficacy.

**Table 2:** Advanced research of biomaterial-based delivery systems in osteosarcoma treatment with therapeutic classification.^a^

Biomaterial carrier	Therapeutic agents	Targeting strategy	Release mechanism	Treatment efficacy	Ref.
Sr²^+^/UV double-crosslinked alginate–GelAGE with PDA particles	DOX	Combined chemotherapy + photothermal therapy	DOX controlled release; PDA-mediated hyperthermia; Sr²^+^ ion release	Kills MG63 osteosarcoma cells via chemo-PTT synergy; promotes rBMSCs proliferation and ALP activity; osteogenic and antitumor dual-function validated *in vitro*	[141]
Gallium-doped bioactive glass	Gallium oxide	Selectively toxic to osteosarcoma cells, sparing normal osteoblasts	Sustained release via dissolution	99% reduction in Saos-2 viability; stimulated apatite layer formation in SBF	[142]
Chitosan-grafted PCL nanofibers loaded with bioactive glasses (BGs)/MBGs	Cisplatin	Local hyperthermia via alternating magnetic field enhances chemotherapy effect on osteosarcoma MG-63 cells	pH 5.5 and 43°C temperature accelerate Cisplatin release	Effective apoptosis/necrosis induction in MG-63 cells *in vitro*; potential for implantable bone tumor therapy device *in vivo*	[143]
Chitosan microspheres	Curcumin and Gallic acid	Positive surface charge facilitates cellular interaction and drug loading	Sustained drug release over ∼7 days in physiological buffer	Negligible toxicity to blood and normal cells; strong anti-osteosarcoma effect on U2OS cells	[144]
Chitosan physically crosslinked with Cu(II) ions	DOX	Synergistic inhibition of tumor growth at lower drug dose; selective toxicity toward osteosarcoma cells	Initial burst release followed by sustained release; pH sensitivity facilitates drug loading and release	Selective toxicity to osteosarcoma MG-63 cells; mild toxicity to healthy human mesenchymal stem cells	[145]
Carboxymethyl chitosan nanoparticles labeled with Na131I	DOX and Curcumin	Anti-EGFR targeting; enhanced tumor penetration; photothermal sensitization via curcumin	Higuchi model-governed sustained release; fractionated dose improves efficacy	513-fold greater targeting efficacy to MG-63 cells; 18.3-fold increased cytotoxicity with fractionated dose; induced G2/M cell cycle arrest	[146]
PLGA and chitosan composite with epoxy-tetrapeptide derivative	Paclitaxel (PTX)	pH-responsive drug release targeting acidic tumor microenvironment	Acidic pH-triggered controlled release	Significant inhibition of osteosarcoma HOS and U2OS cell proliferation; dose-dependent SPICE1 suppression; enhanced drug loading and safety profile	[147]
Tropocollagen grafted with partially deacetylated chitin nanocrystals (CO-g-ChNCs)	Octenidine dihydrochloride	Enhanced cytocompatibility and cell adhesion; designed for skin and bone tissue targeting	Controlled drug release via hierarchical microstructure of layered hydrogel membranes	Improved mechanical properties, cytocompatibility and cell adhesion; potential for controlled drug release and tissue regeneration	[148]
Diopside (CaMgSi_2_O_6_)	Doxorubicin	Bioactive and degradable ceramic scaffold enabling local drug release	Gradual doxorubicin release from scaffold	*In vitro* selective cytotoxicity higher in osteosarcoma MG-63 spheroids vs. primary human fibroblasts	[149]
Aliphatic polyesters with closo-borates in polymer/alginate/gelatin hydrogel composite	Closo-borates for BNCT	Localized boron delivery to tumor cells combined with bone defect repair	Controlled degradation and release of closo-borate ions	High potential for boron neutron capture therapy and bone tissue regeneration	[150]
Chemically crosslinked silk fibroin hydrogel with HRP/H_2_O_2_	Curcumin	Localized delivery via in situ injection	Rapid gelling via HRP/H_2_O_2_-initiated di-tyrosine crosslinking; curcumin-entrapped	Toxic to U2OS osteosarcoma cells; induces rapid apoptosis within 4 hr; β-sheet structure enhanced with curcumin loading	[151]
Chitosan, alginate, hydroxyapatite composite hydrogel	Zoledronic acid	Local bone regeneration scaffold; biocompatible with osteosarcoma cells	Sustained drug release over extended period (48–360 hr)	Biocompatible; increased MG-63 cell viability; porous structure suitable for 3D printing and bone regeneration	[152]
Apatite-infused sol-gel glass	DOX	Osteoinductive + drug reservoir	Mesoporous nanostructure	ALP↑; mineralization↑	[153]
Biodegradable polyelectrolyte complex (PEC) from chitosan + nascent HAp	Ciprofloxacin	Osteoconductive hydroxyapatite combined with biodegradable PEC for targeted bone delivery	Controlled drug release via swelling and degradation kinetics of composite	Cytocompatible with MG-63 and HOS cells; early biomimetic apatite mineralization; antibacterial activity against *S. aureus* and *E. coli in vitro*	[154]
Hierarchically structured HAp microspheres integrated with amphiphilic curcumin prodrug	Curcumin	Selective uptake by osteosarcoma cells; enhanced loading and stability via hierarchical flake-like HA structure	Prodrug self-assembly influenced by hierarchical structure; controlled cargo delivery	Suppressed tumor growth and metastasis; promoted apoptosis; inhibited proliferation and tumor vascularization	[155]
MgO-doped tricalcium phosphate (TCP) scaffolds	Curcumin	Uptake by osteosarcoma cells, intracellular ROS increase via SO2 release	Sustained release of Curcumin (∼22% in 30 days at pH 7.4)	Enhanced bone formation (∼2.5x control), 8.5x osteosarcoma cell viability reduction and 71% antibacterial efficacy *in vivo* rat model	[156]
TCP coated with HAp	Curcumin and Vitamin D3	Bone graft matrix supporting osteogenesis and anticancer activity	Sustained release from CaP matrix and HA coating	2.7-fold increase in new bone formation *in vivo* after 6 weeks; decreased osteosarcoma cell viability *in vitro* after day 11	[157]
Amorphous calcium phosphate and bone-like carbonated nanocrystalline apatite	DOX	Localized delivery; implantable paste for bone defect filling	Controlled local release of doxorubicin from loaded particles	Limited lung metastasis and no signs of toxicity in invasive osteosarcoma rat model	[158]
Octacalcium phosphate	DOX	Osteoconductive + pH-sensitive	Sustained release (6 weeks)	Apoptosis via PARP; MC3T3-E1 proliferation↑	[159]
CaS/HA composite	DOX	Intratumoral depot	Time/pH-sensitive release	Tumor inhibition; enhanced local concentration	[160]
Gelatin/PLA + DOX@nHAp + Icariin	DOX + Icariin	Dual: tumor + bone repair	Burst + sustained release	MG-63 apoptosis; ALP↑; biomineralization↑	[161]
Eggshell-derived hydroxyapatite	Etoposide	Dual: antitumor + antibacterial	Wet-precipitated sustained release	MG-63 apoptosis↑; anti-*S. aureus* activity↑; L929 cytocompatibility	[162]
HAp porous beads (HAPB)	Gentamicin and Vancomycin	Localized delivery via muscle implantation; bioceramic scaffolds for antibiotic release	Sustained antibiotic release with demonstrated biocompatibility	No sub-chronic systemic toxicity observed in rabbits; noncytotoxic to osteosarcoma cells; supports regulatory approval potential	[163]
Poly(L-lactic acid) (PLLA)/nanoscale HAp	Metformin	Combines mechanical strength, bioactivity and water-soluble antitumor MET	Biodegradation of PLLA enables slow sustained MET release	Promoted osteosarcoma cell apoptosis and tumor inhibition *in vitro*; enhanced osteogenic differentiation of BMSCs	[164]
HAp/BSA	Paclitaxel	Tumor targeting via HA; anti-metastasis	Sustained release of PTX and Ca²^+^	Inhibited 143B cell proliferation/migration; promoted osteogenic differentiation in hFOB 1.19	[165]
TCP and PCL composite	Quercetin and potassium chloride (KCl)	Bone tissue engineering scaffold with tunable degradation and drug release	Biphasic diffusion-mediated quercetin release over 28 days	2.1-fold increase in human fetal osteoblast viability; 3-fold reduction in osteosarcoma MG-63 cell viability; antibacterial activity against *S. aureus*	[166]
Mg, Si-doped HAp + sodium alginate + chondroitin sulfate/keratin	Raloxifene hydrochloride	Local delivery to bone via implantable beads	Sustained release over 12 weeks; no burst release; Mg²^+^ crosslinking enhances surface properties	Beads show porous structure, high mechanical stability and prolonged drug release; cytocompatible with osteoblasts, active on OS cells	[167]
β-TCP scaffold	Zoledronic acid (ZOL)	Localized bone-targeted delivery via bound and free ZOL states	Limited release of ZOL; majority bound to scaffold allowing sustained effect	Inhibited osteosarcoma and osteoclast activities; promising novel bone substitute	[168]
Lipid	Alendronate, Cisplatin	Bone homing via bisphosphonate	Controlled lipid release	Low systemic toxicity; effective tumor control	[169]
Polyhydroxybutyrate-co-hydroxyvalerate (PHBV) with mesoporous bioactive glass nanoparticles	Cinnamaldehyde (CIN)	Antibacterial and bioactive; selective activity against *Staphylococcus aureus* and *Escherichia coli*; supports MG-63 proliferation	Sustained cinnamaldehyde release up to 7 days	Antibacterial activity demonstrated; no adverse effect on osteosarcoma MG-63 cell viability; rapid hydroxyapatite formation showing bioactivity	[170]
Mesoporous silica nanoparticles (MSNs) coated with HA	DOX and Selenium (Se)	HA gatekeeper enables pH-responsive intracellular release; dual drug delivery enhances osteosarcoma inhibition	pH-triggered degradation of HA coating allowing controlled release of Dox and Se	Enhanced osteosarcoma inhibitory efficiency *in vitro* for combination therapy vs. single-agent treatments	[171]
Selenium-doped nano-HAp and mesoporous HAp	DOX and Se	Higher selenium doping and Dox loading in n-HA; selective uptake by osteosarcoma cells	Similar Dox release kinetics; selenium release higher in n-HA; sustained release behavior	Potential dual function inhibiting osteosarcoma recurrence and promoting osteogenesis	[172]
Mesoporous silica (Se-doped or Se-coated)	Selenium (SeO_3_²^−^ or SeNP)	ROS induction + osteoblast safety	GSH/NADPH-sensitive release	Selective cytotoxicity (Saos-2↑; osteoblast↓); apoptosis via ROS	[173]
Methylcellulose	Curcumin + IR820	Minimally invasive chemo-PTT	Thermal-triggered gelation	Tumor ablation; ALP↑; MSC osteogenesis↑	[174]
Biodegradable nanocement with Cissus quadrangularis herbal membrane	DOX	Local delivery to tumor resection site; combined tumor cell killing and periosteum/bone formation enhancement	Sustained doxorubicin release over 30 days; herbal membrane supports periosteum and mineralization	2.6-fold increase in bone volume; effective tumor cell killing; periosteum development and mineralized bone callous formation *in vivo*	[175]
CS-SA/CS-FA + Fe_3_O_4_ magnetic nanoparticules	DOX	pH-sensitive + folate receptor targeting	pH-triggered release + magnetic targeting	MG-63 specific uptake and apoptosis; A549 safety profile	[176]
Iron oxide nanoparticles coated with boiling rice starch extract	DOX	Photothermal therapy guided by photoacoustic imaging (PAI); bioactive rice starch coating enhances targeting	pH-dependent drug release in acidic, neutral and basic media; physical immobilization coating of Dox	Excellent photothermal stability and conversion; biocompatible; promising theranostic applications	[177]
Natural melanin nanoparticles from *Sepia officinalis* ink	DOX	Chemo-photothermal therapy triggered by NIR light; biocompatible, cost-effective natural nanoplatform	Sustained drug release enhanced by photothermal stimuli	93% reduction in SaOs-2 osteosarcoma cells viability after 48 hr with NIR; synergistic chemo-photothermal antitumor effect	[178]
LDH/PCL	Alendronate	Controlled release via LDH intercalation in PCL	Sustained release enhanced with 15 wt% LDH	Increased MG-63 viability, attachment and mineralization; stable ALP activity	[179]
Zr-HA + modified PCL + β-CD	Cisplatin + Curcumin	Dual spatial/temporal targeting	Porosity + inclusion complex	Tumor suppression; osteoinduction↑	[180]
PCL composite films modified with BG particles	Polyphenols extracted from *Salvia officinalis* L.	BG particles modulate polyphenol release kinetics and polyphenol-binding capacity for selective osteosarcoma cytotoxicity	Controlled polyphenol release influenced by BG texture and surface chemistry	Selective cytotoxicity and antiproliferative effect on osteosarcoma Saos-2 cells; no toxicity to normal osteoblasts	[181]
HEMA/MMA + phosphate coating	DOX	pH-responsive + osteogenic	Degradation + acid-triggered release	MG-63 apoptosis; post-treatment CaP formation	[182]
PLGA + PEG4000 implant	DOX	Local depot delivery	Three-phase release profile	Local drug accumulation↑; systemic toxicity↓	[183]
PLGA core + 143B-RAW hybrid membrane	Paclitaxel	Tumor homing + immune evasion	Sustained release + chemotaxis	Xenograft inhibition; low off-target toxicity	[184]
mPEG-b-P(C7-co-CA) amphiphilic polymeric prodrug micelles	Cinnamaldehyde	pH-triggered charge conversion (neutral to positive) targets acidic osteosarcoma environment (pH 6.5)	pH-dependent drug release via polymer micelle destabilization	High *in vitro* anti-osteosarcoma efficacy; increased ROS generation and apoptosis in 143B cells	[185]
Polymethylmethacrylate (PMMA) cement with CMC additive	Cisplatin	Porosity introduced for localized cisplatin delivery while maintaining mechanical strength	Sustained release of cisplatin (∼18% over 28 days)	Efficient osteosarcoma cell killing *in vitro*; compressive strength comparable to intact tibias; viable for load-bearing bone defect treatment	[186]
Polydopamine (PDA)-modified silk fibroin (SF) composite scaffold	Curcumin	Localized delivery combining photothermal effect and chemotherapy for synergistic osteosarcoma targeting	Low pH and NIR irradiation accelerate CM release	Long-term stable anticancer effect; enhanced osteosarcoma MG-63 inhibition under NIR; promotes osteoblast proliferation	[187]
Bacterial exopolysaccharide	Curcumin	pH-responsive release targeting acidic tumor microenvironment	pH-dependent release, higher release at acidic pH	Cytotoxicity against osteosarcoma MG-63 cells; superior stability, hemocompatibility, antibacterial, antibiofilm and antioxidant activity	[188]
PEG	DOX	PEG reduces protein adsorption and cardiotoxicity; UV-triggered PEG detachment enhances uptake	UV-induced cleavage of amide bond detaches PEG, increasing Dox cellular uptake	No significant organ toxicity; efficient tumor suppression observed in osteosarcoma models	[189]
Chondroitin sulfate (marine-derived polysaccharide)	N/A (nanoparticles themselves therapeutic)	Biocompatible marine polysaccharide coating enhances nanoparticle stability and delivery	Passive uptake, no drug release per se	Selective cytotoxicity against osteosarcoma MG63 cells by ROS-mediated mitochondrial damage; apoptosis activation confirmed by flow cytometry	[190]
Amphiphilic polymer (mPEG-P(HDI-DN))	Sulfur dioxide (SO_2_) gas therapy agent	–	GSH-triggered degradation of polymer releasing SO_2_ gas	Effective osteosarcoma growth inhibition without obvious tissue toxicity *in vivo*	[191]
Bioglass + PVA + Cellulose (CNC) + SA	Methotrexate (MTX)	Localized delivery	Diffusion + Bioglass-tuned	MG-63 cytotoxicity; sustained release	[192]
Silk fibroin + nHA + Curcumin-modified PDA	Curcumin	Chemo-PTT + bone regeneration	Photothermal + sustained	Tumor suppression + *in vivo* bone regeneration	[193]
Alendronate-conjugated polyethylene glycol functionalized chitosan (ALD-PEG-CHI)	Cell death siRNA (CD-siRNA) and Curcumin (CUR)	Bone targeting via alendronate conjugation; synergistic cytotoxicity of siRNA and CUR delivery	Encapsulation and controlled release; size < 200 nm; near-neutral zeta potential	*In vitro* synergistic growth inhibition of MCF-7 cells; 5-fold enhanced bone targeting compared to controls	[194]
PLGA + Chitosan core + pectin/chitosan shell	miRNA-34a + Dox + Resveratrol	Gene + chemo therapy	Layer-by-layer release	U2OS/Saos-2 apoptosis ↑; synergistic effect	[195]
Chitosan modified with cell-penetrating peptide (H6R6), thermosensitive hydrogel	Doxorubicin and siRad18 siRNA	Enhanced tumor permeability via peptide-modified NPs; siRNA-mediated gene silencing to overcome Dox resistance; combined with PD-L1 immune checkpoint blockade	Controlled nanoparticle release from thermosensitive hydrogel	Significant inhibition of osteosarcoma growth and lung metastasis *in vivo*; enhanced immunogenic cell death; improved chemo- and immunotherapy efficacy	[53]
Hyaluronic acid hydrogel + nanoparticles	microRNA-29b	Localized delivery to osteosarcoma orthotopic tumor site; combined with systemic chemotherapy	Sustained local release via hydrogel matrix	Reduced tumor burden, increased survival, decreased osteolysis and normalized bone homeostasis *in vivo*	[196]
Alginate-based hydrogel with hyaluronic acid and gelatin	Reactive Oxygen and Nitrogen Species generated from Cold Atmospheric Plasma	Dual action: selective cytotoxic anticancer agent against osteosarcoma and promoter of stem cell-mediated bone regeneration	Controlled and localized delivery of plasma-derived RONS within hydrogel	Selective cytotoxicity against osteosarcoma MG-63 cells; promotion of human mesenchymal stem cells proliferation and osteogenic differentiation	[197]
Hybrid tumor and macrophage membranes coated on PLGA nanoparticles	microRNA-665	Tumor and macrophage membrane camouflage for tumor targeting; modulation of tumor microenvironment macrophages	Nanoparticle encapsulation; targeted delivery via membrane cloaking	Promoted M1 macrophage polarization, inhibited osteosarcoma MG-63 proliferation and migration; significant tumor growth inhibition *in vivo*; good biosafety	[198]
CO_2_-derived cationic poly(vinylcyclohexene carbonates)	siRNA targeting PLK1	Gene silencing of PLK1; biodegradable nonviral vector	Endosomal escape and gene transfection	Induced G2/M arrest and apoptosis; significant tumor regression *in vitro* and *in vivo*	[116]
Mg^2+^-releasing hydrogel	Anti-PD-L1 + Vismodegib + Mg^2+^	Immunotherapy + bone regeneration	Sustained release	Tumor suppression; osteogenic gene upregulation	[199]
Thermosensitive and zwitterionic polymer + mesoporous nanoparticles	Immune Checkpoint Blockade (ICB) agents	Local delivery to surgical site; thermogel prevents early leakage and increases tumor-site drug accumulation	NIR-induced heat triggers retro Diels–Alder reaction to degrade nanoparticle coating, enabling controlled ICB release	Effective prevention of osteosarcoma recurrence; enhanced T cell activation; favorable biocompatibility observed	[200]

a This table compiles 64 representative original research articles on biomaterial-based delivery systems applied in osteosarcoma treatment from 2021 to 2025. This classification facilitates comparative analysis of biomaterial platforms and their associated therapeutic strategies in osteosarcoma research and development.

## Biomaterials for bone regeneration in osteosarcoma treatment

**Figure 6:**
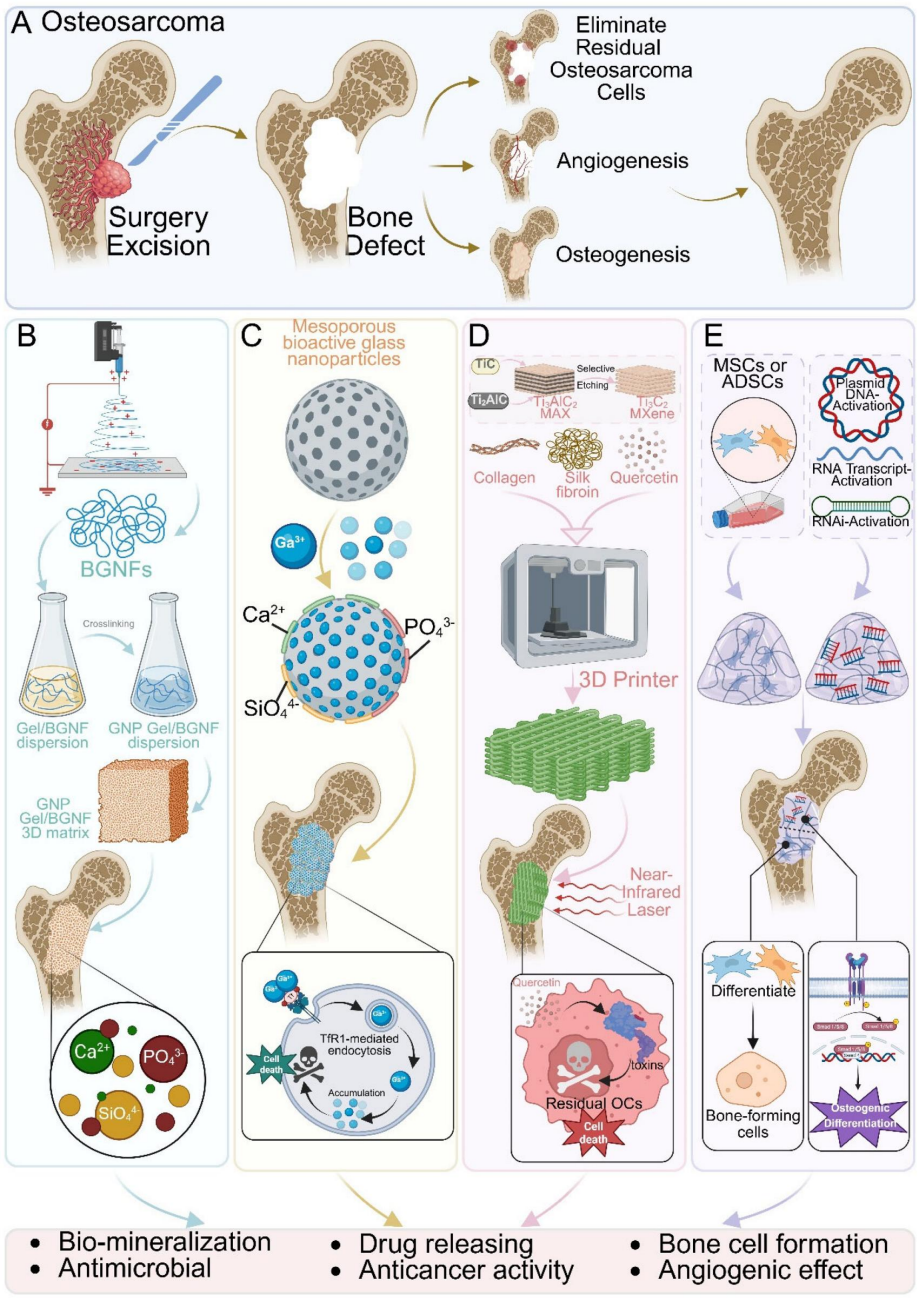
Schematic illustration of integrated bone tissue engineering strategies for osteosarcoma. This figure outlines multifunctional biomaterial approaches designed to address both tumor eradication and post-resection bone defect repair following osteosarcoma surgery. (**A**) After surgical excision of the tumor, scaffolds and therapeutics aim to eliminate residual osteosarcoma cells while promoting angiogenesis and osteogenesis to restore bone integrity. (**B**) BGNF-based 3D matrices fabricated via crosslinking of bioactive glass nanofibers and gelatin provide structural support and stimulate biomineralization, with intrinsic antimicrobial properties. (**C**) Mesoporous bioactive glass nanoparticles, doped with ions such as Ga^3+^, Ca^2+^ and ${\text{PO}}_{4}^{3-}$, enable targeted drug release and anticancer effects through TfR1-mediated endocytosis, inducing tumor cell death. (**D**) 3D-printed composite scaffolds incorporating materials such as MXene, silk fibroin, collagen and quercetin enable photothermal activation under near-infrared light to selectively ablate tumor cells while supporting osteogenic regeneration. (**E**) Gene-activated scaffolds and stem cell-based constructs, using MSCs or ADSCs combined with plasmid DNA, RNA activation systems or signaling molecules, promote bone-forming cell differentiation and angiogenesis through localized gene expression. These strategies combine biochemical, biophysical and cellular interventions to create a regenerative microenvironment that supports bone healing while suppressing tumor recurrence. Created with BioRender.com.

While the previous discussion focused on biomaterials for tumor-targeted therapies, another essential goal in osteosarcoma management is the restoration of bone integrity after surgical resection. The treatment of osteosarcoma presents a unique challenge in the field of bone tissue engineering, as it requires not only the elimination of residual tumor cells but also the promotion of bone regeneration in the affected area (Figure 6). Recent advancements in biomaterials and cell-based therapies have shown promising results in addressing these dual needs, offering new hope for improved patient outcomes.

### Bioactive 3D scaffolds: promoting bone cell proliferation postsurgery

Three-dimensional scaffolds play a crucial role in bone tissue engineering by providing a structural framework that mimics the natural extracellular matrix, supporting cell adhesion, proliferation and differentiation [201]. In the context of osteosarcoma treatment, these scaffolds serve as temporary substitutes for the removed bone tissue, guiding the regeneration process [202]. The development of multifunctional nanofibrous 3D matrices has shown particular promise, combining the benefits of tissue engineering with photothermal therapy for postoperative treatment of osteosarcoma [203].

One innovative approach involves the use of flexible bioactive glass nanofibers (BGNFs) as the basic building blocks of these scaffolds [204]. When combined with genipin-crosslinked gelatin (GNP-Gel), these materials form stable 3D structures that closely resemble the extracellular matrix. The porous nature of these scaffolds, achieved through ice crystal templating and freeze-drying techniques, allows for efficient cell infiltration and nutrient transport. Moreover, the excellent compression recovery performance of these matrices in water makes them suitable for minimally invasive surgical applications, a significant advantage in postoperative care.

The incorporation of bioactive materials such as HAp and bioactive glass into scaffold designs has revolutionized bone tissue engineering [205]. These materials not only provide structural support but also actively stimulate bone cell proliferation and differentiation, accelerating the healing process. Hydroxyapatite-tricalcium phosphate (HAP-TCP) bioceramics have demonstrated remarkable osteoinductive and osteointegrative properties in experimental studies. When used to fill metaphyseal defects, these materials promote a dynamic and uncomplicated course of reparative osteogenesis [206]. The gradual arrangement of osteoclasts and osteoblasts on the surface of HAP-TCP implants indicates high biocompatibility with bone tissue, facilitating the processes of implant resorption, mineralization and the formation of mature bone tissue [207]. Bioactive glasses, particularly those containing boron (e.g. 13-93B20), have shown enhanced reactivity compared to traditional silicate glasses [208, 209]. Hybrid scaffolds combining these bioactive glass particles with gelatin exhibit faster initial dissolution and more rapid precipitation of a HAp layer, which is crucial for bone bonding and regeneration. The ability to tailor glass composition allows for better control over material dissolution, biodegradability and bioactivity, making these hybrids promising candidates for bone applications in osteosarcoma treatment.

The development of multifunctional biomaterials that can simultaneously address cancer treatment and bone regeneration represents a significant advancement in osteosarcoma therapy. Gallium-doped bioactive glasses have shown promising results in this regard, demonstrating the ability to selectively kill human osteosarcoma cells while promoting excellent *in vivo* osteointegration [210]. Studies have shown that cell culture media conditioned with gallium-doped bioactive material can kill up to 41% of osteosarcoma cells without significantly affecting normal human osteoblasts. Furthermore, *in vivo* experiments have demonstrated excellent material-bone integration with no signs of local toxicity or implant rejection. This approach offers the potential for synergistic bone regeneration and targeted cancer therapy, paving the way for new bone cancer treatment strategies that address both tumor elimination and tissue regeneration in a single material.

Recent developments in scaffold design have focused on creating structures that more closely mimic the complex architecture of natural bone [211]. This biomimetic approach enhances bone healing post-tumor removal by providing an environment that closely resembles the native tissue. Notable advancements include the development of MXene-integrated (Ti_3_C_2_) silk fibroin-based self-assembly-driven 3D-printed scaffolds, which offer combined photothermal tumor ablation and osteogenic support, as well as silk fibroin/polyacrylamide-based tough 3D-printed scaffolds with strain-sensing capacity and chondrogenic activity, demonstrating the versatility of silk fibroin in multifunctional scaffold engineering [212, 213]. These theragenerative scaffolds combine the benefits of a controlled pore size and macroscopic geometry with mechanical stability. The integration of MXene two-dimensional nanosheets into the scaffold structure endows it with a remotely controlled photothermal anti-osteosarcoma ablation function. This dual functionality allows for both the elimination of residual cancer cells through photothermal therapy and the stimulation of bone mineral deposition on the scaffold surface.

### Cell-based and gene-based therapies: enhancing bone repair

The incorporation of stem cells and osteoblasts into biomaterial scaffolds has emerged as a powerful strategy to enhance bone repair in osteosarcoma cases [214]. Mesenchymal stem cells (MSCs), in particular, have shown great promise due to their ability to differentiate into bone-forming cells and their secretion of bioactive factors that promote tissue regeneration [215]. Recent advancements in biomaterials have focused on creating scaffolds that not only support but also actively promote stem cell differentiation [216]. These scaffolds are designed to provide the necessary physical and biochemical cues to guide MSCs towards an osteogenic lineage. For example, the combination of MSCs with biocompatible scaffolds has been shown to improve cell tracking and retention at the site of injury, addressing one of the major challenges in cell-based therapies [217]. Adipose-derived stem cells (ADSCs) have gained increasing attention as an alternative to bone marrow-derived MSCs due to their abundant sources, easy availability and multidifferentiation potential [218]. The selection of appropriate biomaterials to be combined with ADSCs is crucial for optimizing their therapeutic effect. Factors such as biocompatibility, inflammation regulation, angiogenesis promotion and osteogenesis induction are key considerations in designing ADSC-loaded scaffolds for bone regeneration in osteosarcoma treatment [219]. In addition to cell-loaded scaffolds, immunomodulatory scaffolds have gained increasing attention for their ability to simultaneously support bone regeneration and suppress tumor recurrence. For example, GelMA-based hydrogels loaded with STING agonists or anti-PD-L1 antibodies can activate local immune responses while promoting osteoblast activity and scaffold integration. Mineralized scaffolds incorporating immunogenic peptides or cytokine-mimicking nanoparticles have also been explored to recruit antigen-presenting cells and remodel the local immune microenvironment in favor of osteogenesis. These dual-function scaffolds represent a promising direction for osteosarcoma treatment, integrating immunotherapeutic potential with regenerative performance.

In bone tissue engineering, to avoid the side effects of inflammation, potential tumorigenicity and ectopic osteogenesis caused by high-dose growth factor administration, researchers have begun to turn to gene therapy and RNA interference (RNAi) technology [220]. Although traditional protein drugs (such as BMP, VEGF, FGF, etc.) can promote osteogenesis and angiogenesis, they are easily degraded and diluted in the body, and require repeated or high-dose administration, which brings a series of safety risks [221]. In contrast, gene and RNAi vectors are transfected/transduced in local cells, so that the cells themselves stably express (or inhibit) the target protein, which can not only maintain long-term efficacy but also reduce side effects caused by high-dose proteins. The two most widely used gene delivery methods are viral vectors (high transduction efficiency but with immune and insertion mutation risks) and nonviral vectors (high safety but low transgenic efficiency) [222]. Combining these vectors with scaffold materials, including gene-activated scaffolds and RNAi-activated scaffolds, they can *in situ* transduce/transfect host cells after transplantation into the body, thereby inducing the expression of target genes or inhibiting the expression of specific genes [223]. Compared with the method of culturing and genetically modifying cells before transplantation and then implanting them into scaffolds, gene-activated scaffolds are expected to become “ready-made” bone repair alternatives that do not require cell culture operations, reducing problems such as differences in cell sources, cell transportation and operational difficulties. The feasibility and safety of gene therapy have been demonstrated in bone tissue engineering, which can provide ideas for the treatment of malignant bone tumors such as osteosarcoma. On the one hand, gene vectors are used to continuously express proteins that inhibit tumor proliferation or metastasis in the local microenvironment of the tumor; on the other hand, RNAi technology can downregulate the expression of genes related to osteosarcoma growth [224]. The combination of the two may achieve a more lasting and low-toxicity therapeutic effect at both the bone repair and antitumor levels.

In conclusion, the field of bone tissue engineering for osteosarcoma treatment has made significant strides in recent years, with the development of advanced biomaterials and cell-based therapies offering new possibilities for improved patient outcomes. The integration of 3D scaffolds, bioactive materials and stem cell technologies provides a multifaceted approach to addressing the complex challenges of bone regeneration in the context of cancer treatment. As research continues to advance, these innovative strategies hold great promise for enhancing the efficacy of osteosarcoma treatment and improving the quality of life for patients facing this challenging condition.

## Challenge and perspective

Among the diverse biomaterial strategies reviewed, some platforms appear particularly promising for clinical translation in osteosarcoma treatment. Multifunctional composite scaffolds that integrate tumor ablation, immune modulation and bone regeneration stand out for their ability to address multiple therapeutic goals simultaneously. For example, Ti_3_C_2_-MXene-integrated silk fibroin scaffolds have demonstrated both photothermal antitumor activity and osteoinductive potential, highlighting their value in post-resection therapy.

Hydrogel-based systems, due to their injectability, tunable degradation profiles and compatibility with various therapeutic payloads, also show strong adaptability across drug, gene and immunotherapy delivery contexts. In contrast, immune-responsive and gene-activated scaffolds, while conceptually advanced, remain mostly in early-stage development and face greater translational hurdles, including delivery precision and preclinical validation in bone tumor models.

Biomaterial-based strategies for osteosarcoma therapy face significant challenges that span from immunological responses to the complexities of the tumor microenvironment. On one hand, implanted biomaterials often trigger immune reactions, such as inflammation, encapsulation or rejection, that compromise therapeutic efficacy and potentially worsen the tumor microenvironment [225, 226]. Surface modification techniques and the integration of specific metal dopants can help modulate the immune response by creating more biocompatible interfaces [227, 228]. On the other hand, the harsh osteosarcoma microenvironment, characterized by hypoxia, acidosis and high interstitial fluid pressure, poses additional barriers to drug delivery and tissue regeneration [229]. Overcoming these obstacles requires biomaterials capable of adaptive or smart responses, such as engineered extracellular vesicles or nanoparticles designed to release therapeutic agents in direct response to local environmental cues [230–232].

In addition to biological and design-related challenges, the translation of biomaterials into clinical practice presents significant hurdles. Regulatory approval requires comprehensive assessment of sterility, safety and long-term performance, all of which vary by material class and region. Many complex or multifunctional biomaterials also face obstacles in large-scale, GMP-compliant production, owing to batch variability or bioactivity loss during processing. Sterilization techniques such as gamma irradiation or ethylene oxide can damage sensitive therapeutic components, and maintaining functional stability during storage and transportation remains difficult. To address these challenges, researchers are exploring bioactivity-preserving sterilization methods (e.g. supercritical CO_2_ or aseptic lyophilization), cryopreservation-compatible scaffolds and modular manufacturing approaches aligned with regulatory frameworks. Early collaboration with regulatory agencies and the adoption of standardized protocols for material characterization and quality control will be key to accelerating clinical translation of biomaterial-based therapies for osteosarcoma.

Looking ahead, multiple innovative directions hold promise for enhancing osteosarcoma treatments. Smart nanomaterials, like dendrimers, nanogels and stimulus-responsive polymers, can target osteosarcoma more precisely and deliver therapeutics with spatiotemporal control [18, 233]. Personalized biomaterial scaffolds, tailored to patients’ genomic and proteomic profiles, may further optimize therapeutic outcomes by matching individual tumor characteristics and bone structures [234, 235]. Additionally, multifunctional and hybrid systems, such as 2D mesoporous silica@MXene or Ti6Al4V-based composites, illustrate how diagnosis and therapy can be combined within a single platform to ablate cancer cells while encouraging bone regeneration [236, 237]. The integration of inorganic components, like black phosphorous, magnesium, zinc, copper, silver, into responsive biomaterials also shows potential for improved biodegradation, antibacterial activity and tumor suppression [238, 239]. While these emerging strategies collectively represent exciting progress, some appear particularly well-positioned for near-term clinical translation. Multifunctional composite scaffolds, capable of simultaneously supporting tumor suppression, immune modulation and bone regeneration, are among the most promising, due to their integrative design and alignment with surgical practice. Similarly, microenvironment-adaptive systems, such as pH- or hypoxia-responsive hydrogels, offer improved therapeutic specificity while minimizing off-target effects. In contrast, although personalized scaffolds based on omics data or patient-derived structures represent a compelling vision for the future, their widespread application is currently constrained by challenges in cost, standardization and regulatory acceptance. Likewise, diagnostics, therapeutic hybrid platforms, though technologically sophisticated, may require further simplification for scalable and robust deployment. Achieving these goals will require close collaboration among materials scientists, immunologists and clinicians, as well as partnerships between academia, industry and healthcare providers to navigate technical, regulatory and scalability challenges. Through continued interdisciplinary innovation, biomaterials stand to transform osteosarcoma treatment and offer new hope to patients facing this aggressive disease. Therefore, materials that balance functional complexity with manufacturability, safety and translational feasibility are likely to lead the next phase of innovation in osteosarcoma biomaterials.

## Conclusion

This comprehensive review highlights the transformative potential of biomaterials in osteosarcoma treatment across multiple therapeutic domains. This work stands out for its comprehensive coverage of diverse biomaterial applications in osteosarcoma treatment. It uniquely integrates insights from drug delivery, gene therapy and bone regeneration, providing a holistic view of the field. The review’s novel points include the emphasis on multifunctional biomaterials that combine therapeutic delivery with diagnostic capabilities, the exploration of nature-inspired delivery systems like leukosomes and calcium-modified diatoms, and the detailed discussion of advanced scaffold designs that mimic bone architecture. Additionally, this review introduces the emerging class of smart or stimuli-responsive biomaterials, which respond to tumor-specific cues and offer new avenues for precision-controlled therapy. A summary of 64 recent studies is provided to compare biomaterial-based delivery strategies, emphasizing the promise of multifunctional and composite systems in osteosarcoma treatment.

Despite these advances, several challenges remain, including material immunogenicity, delivery efficiency in the complex tumor microenvironment and regulatory barriers to clinical translation. Future research should prioritize the development of microenvironment-adaptive materials, personalized scaffolds tailored to tumor and bone profiles and clinically scalable fabrication methods. Collaborative efforts across materials science, oncology and translational medicine will be essential to bridge the gap between laboratory innovation and patient-centered application.

Overall, this review offers not only a comprehensive summary of current strategies but also a roadmap for designing next-generation biomaterials capable of reshaping the therapeutic landscape of osteosarcoma.

## References

[1] Beird HC , Bielack SS, Flanagan AM, Gill J, Heymann D, Janeway KA, Livingston JA, Roberts RD, Strauss SJ, Gorlick R. Osteosarcoma. Nat Rev Dis Primers 2022;8:77. 36481668 10.1038/s41572-022-00409-y

[2] Jiang Y , Wang J, Sun M, Zuo D, Wang H, Shen J, Jiang W, Mu H, Ma X, Yin F, Lin J, Wang C, Yu S, Jiang L, Lv G, Liu F, Xue L, Tian K, Wang G, Zhou Z, Lv Y, Wang Z, Zhang T, Xu J, Yang L, Zhao K, Sun W, Tang Y, Cai Z, Wang S, Hua Y. Multi-omics analysis identifies osteosarcoma subtypes with distinct prognosis indicating stratified treatment. Nat Commun 2022;13:7207. 36418292 10.1038/s41467-022-34689-5 PMC9684515

[3] Gill J , Gorlick R. Advancing therapy for osteosarcoma. Nat Rev Clin Oncol 2021;18:609–24. 34131316 10.1038/s41571-021-00519-8

[4] Cole S , Gianferante DM, Zhu B, Mirabello L. Osteosarcoma: a surveillance, epidemiology, and end results program-based analysis from 1975 to 2017. Cancer 2022;128:2107–18. 35226758 10.1002/cncr.34163 PMC11647566

[5] Bielack S , Cable MG, Gorlick R, Hecker-Nolting S, Kager L, Marina N, Randall RL, Whelan J. Osteosarcoma-approach to therapy. In: Arndt CAS (ed). Sarcomas of Bone and Soft Tissues in Children and Adolescents. Cham: Springer International Publishing, 2021, 91–109.

[6] Zhao X , Wu Q, Gong X, Liu J, Ma Y. Osteosarcoma: a review of current and future therapeutic approaches. Biomed Eng Online 2021;20:24. 33653371 10.1186/s12938-021-00860-0 PMC7923306

[7] Harris MA , Hawkins CJ. Recent and ongoing research into metastatic osteosarcoma treatments. Int J Mol Sci 2022;23:3817. 35409176 10.3390/ijms23073817 PMC8998815

[8] Halalsheh H , Amer S, Sultan I. Progression before local control in osteosarcoma: outcome and prognosis-predictive factors. Pediatr Blood Cancer 2023;70:e30649. 37638816 10.1002/pbc.30649

[9] Gazouli I , Kyriazoglou A, Kotsantis I, Anastasiou M, Pantazopoulos A, Prevezanou M, Chatzidakis I, Kavourakis G, Economopoulou P, Kontogeorgakos V, Papagelopoulos P, Psyrri A. Systematic review of recurrent osteosarcoma systemic therapy. Cancers (Basel) 2021;13:1757. 33917001 10.3390/cancers13081757 PMC8067690

[10] Pilavaki P , Gahanbani Ardakani A, Gikas P, Constantinidou A. Osteosarcoma: current concepts and evolutions in management principles. J Clin Med 2023;12:2785. 37109122 10.3390/jcm12082785 PMC10143544

[11] Manavi MA , Fathian Nasab MH, Mohammad Jafari R, Dehpour AR. Mechanisms underlying dose-limiting toxicities of conventional chemotherapeutic agents. J Chemother 2024;36:623–53. 38179685 10.1080/1120009X.2023.2300217

[12] Hattinger CM , Patrizio MP, Fantoni L, Casotti C, Riganti C, Serra M. Drug resistance in osteosarcoma: emerging biomarkers, therapeutic targets and treatment strategies. Cancers (Basel) 2021;13:2878. 34207685 10.3390/cancers13122878 PMC8228414

[13] Ren X , Chen X, Geng Z, Su J. Bone-targeted biomaterials: strategies and applications. Chem Eng J 2022;446:137133.

[14] Li J , Zhang H, Han Y, Hu Y, Geng Z, Su J. Targeted and responsive biomaterials in osteoarthritis. Theranostics 2023;13:931–54. 36793867 10.7150/thno.78639 PMC9925319

[15] Harun-Or-Rashid M , Aktar MN, Hossain MS, Sarkar N, Islam MR, Arafat ME, Bhowmik S, Yusa S-I. Recent advances in micro- and nano-drug delivery systems based on natural and synthetic biomaterials. Polymers (Basel) 2023;15:4563. 38231996 10.3390/polym15234563 PMC10708661

[16] Sharma S , Sudhakara P, Singh J, Ilyas RA, Asyraf MRM, Razman MR. Critical review of biodegradable and bioactive polymer composites for bone tissue engineering and drug delivery applications. Polymers (Basel) 2021;13:2623. 34451161 10.3390/polym13162623 PMC8399915

[17] Joyce K , Fabra GT, Bozkurt Y, Pandit A. Bioactive potential of natural biomaterials: identification, retention and assessment of biological properties. Signal Transduct Target Ther 2021;6:122. 33737507 10.1038/s41392-021-00512-8 PMC7973744

[18] Feng C , Jiang Y, Wang T, Tian D, Shen C, Wang Y, Qian H. Recent advances on nanostructured biomaterials in osteosarcoma treatment. Coord Chem Rev 2023;493:215315.

[19] Han X , Alu A, Liu H, Shi Y, Wei X, Cai L, Wei Y. Biomaterial-assisted biotherapy: a brief review of biomaterials used in drug delivery, vaccine development, gene therapy, and stem cell therapy. Bioact Mater 2022;17:29–48. 35386442 10.1016/j.bioactmat.2022.01.011 PMC8958282

[20] Aggarwal D , Kumar V, Sharma S. Drug-loaded biomaterials for orthopedic applications: a review. J Control Release 2022;344:113–33. 35240227 10.1016/j.jconrel.2022.02.029

[21] Wang Y , Zhang H, Qiang H, Li M, Cai Y, Zhou X, Xu Y, Yan Z, Dong J, Gao Y, Pan C, Yin X, Gao J, Zhang T, Yu Z. Innovative biomaterials for bone tumor treatment and regeneration: tackling postoperative challenges and charting the path forward. Adv Healthc Mater 2024;13:e2304060. 38429938 10.1002/adhm.202304060

[22] Suhag D. Future perspectives. In: Suhag D (ed). Handbook of Biomaterials for Medical Applications, Volume 2: Applications. Singapore: Springer Nature Singapore, 2024, 373–89.

[23] Kortam S , Lu Z, Zreiqat H. Recent advances in drug delivery systems for osteosarcoma therapy and bone regeneration. Commun Mater 2024;5:168.

[24] Shoaib Z , Fan TM, Irudayaraj JMK. Osteosarcoma mechanobiology and therapeutic targets. Br J Pharmacol 2022;179:201–17. 34679192 10.1111/bph.15713 PMC9305477

[25] Cojocaru F-D , Balan V, Verestiuc L. Advanced 3D magnetic scaffolds for tumor-related bone defects. Int J Mol Sci 2022;23:16190. 36555827 10.3390/ijms232416190 PMC9788029

[26] Salthouse D , Novakovic K, Hilkens CMU, Ferreira AM. Interplay between biomaterials and the immune system: challenges and opportunities in regenerative medicine. Acta Biomater 2023;155:1–18. 36356914 10.1016/j.actbio.2022.11.003

[27] Tripathi AS , Zaki MEA, Al-Hussain SA, Dubey BK, Singh P, Rind L, Yadav RK. Material matters: exploring the interplay between natural biomaterials and host immune system. Front Immunol 2023;14:1269960. 37936689 10.3389/fimmu.2023.1269960 PMC10627157

[28] Henriksen K , Karsdal MA. Chapter 1- Type I collagen. In: Karsdal MA (ed). Biochemistry of Collagens, Laminins and Elastin, 3rd edn. London, UK: Academic Press, 2024;1–11.

[29] Zhu X , Wang C, Bai H, Zhang J, Wang Z, Li Z, Zhao X, Wang J, Liu H. Functionalization of biomimetic mineralized collagen for bone tissue engineering. Mater Today Bio 2023;20:100660. 10.1016/j.mtbio.2023.100660 PMC10199226 37214545

[30] Jirofti N , Hashemi M, Moradi A, Kalalinia F. Fabrication and characterization of 3D printing biocompatible crocin-loaded chitosan/collagen/hydroxyapatite-based scaffolds for bone tissue engineering applications. Int J Biol Macromol 2023;252:126279. 37572811 10.1016/j.ijbiomac.2023.126279

[31] Qin W , Li C, Liu C, Wu S, Liu J, Ma J, Chen W, Zhao H, Zhao X. 3D printed biocompatible graphene oxide, attapulgite, and collagen composite scaffolds for bone regeneration. J Biomater Appl 2022;36:1838–51. 35196910 10.1177/08853282211067646

[32] Moreno GH. A Cold Plasma-Enabled Reduction Process for the Fabrication of Metallic Nanostructures Onto Polymeric Biomaterials. Alabama: The University of Alabama at Birmingham, 2023, 198.

[33] Zhu M , Zhang R, Mao Z, Fang J, Ren F. Topographical biointerface regulating cellular functions for bone tissue engineering. Biosurf Biotribol 2022;8:165–87.

[34] Qin D , Wang N, You XG, Zhang AD, Chen XG, Liu Y. Collagen-based biocomposites inspired by bone hierarchical structures for advanced bone regeneration: ongoing research and perspectives. Biomater Sci 2022;10:318–53. 34783809 10.1039/d1bm01294k

[35] Pellegrini E , Desando G, Petretta M, Cellamare A, Cristalli C, Pasello M, Manara MC, Grigolo B, Scotlandi K. A 3D collagen-based bioprinted model to study osteosarcoma invasiveness and drug response. Polymers (Basel) 2022;14:4070. 36236019 10.3390/polym14194070 PMC9571197

[36] Zhang Y , Wang J. Current status and prospects of gelatin and its derivatives in oncological applications: review. Int J Biol Macromol 2024;274:133590. 38996884 10.1016/j.ijbiomac.2024.133590

[37] Hassan MA , Basha AA, Eraky M, Abbas E, El-Samad LM. Advancements in silk fibroin and silk sericin-based biomaterial applications for cancer therapy and wound dressing formulation: a comprehensive review. Int J Pharm 2024;662:124494. 39038721 10.1016/j.ijpharm.2024.124494

[38] Harishchandra Yadav R , Kenchegowda M, Angolkar M, T S M, Ali M. Osmani R, Palaksha S, Veerabhadrappa Gangadharappa H. A review of silk fibroin-based drug delivery systems and their applications. Eur Polym J 2024;216:113286.

[39] Yu B , Li Y, Lin Y, Zhu Y, Hao T, Wu Y, Sun Z, Yang X, Xu H. Research progress of natural silk fibroin and the application for drug delivery in chemotherapies. Front Pharmacol 2022;13:1071868. 36686706 10.3389/fphar.2022.1071868 PMC9845586

[40] Liang W , Long H, Zhang H, Bai J, Jiang B, Wang J, Fu L, Ming W, Zhao J, Zeng B. Bone scaffolds-based localized drugs delivery for osteosarcoma: current status and future perspective. Drug Deliv 2024;31:2391001. 39239763 10.1080/10717544.2024.2391001 PMC11382735

[41] Yao X , Zou S, Fan S, Niu Q, Zhang Y. Bioinspired silk fibroin materials: from silk building blocks extraction and reconstruction to advanced biomedical applications. Mater Today Bio 2022;16:100381. 10.1016/j.mtbio.2022.100381 PMC9395666 36017107

[42] Zhu T , Cai G, Zhao W, Yao X, Zhang Y. Effects of silk fibroin hydrogel degradation on the proliferation and chondrogenesis of encapsulated stem cells. Biomacromolecules 2025;26:1305–19. 39842034 10.1021/acs.biomac.4c01676

[43] Liu X , Ouyang Q, Yao X, Zhang Y. A facile nanopattern modification of silk fibroin electrospun scaffold and the corresponding impact on cell proliferation and osteogenesis. Regen Biomater 2024;11:rbae117. 39575301 10.1093/rb/rbae117 PMC11580685

[44] Pham DT , Ha TKQ, Nguyen MQ, Tran VD, Nguyen VB, Quyen TTB. Silk fibroin nanoparticles as a versatile oral delivery system for drugs of different biopharmaceutics classification system (BCS) classes: a comprehensive comparison. J Mater Res 2022;37:4169–81.

[45] Pirota V , Bisbano G, Serra M, Torre ML, Doria F, Bari E, Paolillo M. cRGD-functionalized silk fibroin nanoparticles: a strategy for cancer treatment with a potent unselective naphthalene diimide derivative. Cancers (Basel) 2023;15:1725. 36980611 10.3390/cancers15061725 PMC10046852

[46] Zou S , Yao X, Shao H, Reis RL, Kundu SC, Zhang Y. Nonmulberry silk fibroin-based biomaterials: impact on cell behavior regulation and tissue regeneration. Acta Biomater 2022;153:68–84. 36113722 10.1016/j.actbio.2022.09.021

[47] Ashique S , Faiyazuddin M, Afzal O, Gowri S, Hussain A, Mishra N, Garg A, Maqsood S, Akhtar MS, Altamimi ASA. Advanced nanoparticles, the hallmark of targeted drug delivery for osteosarcoma-an updated review. J Drug Deliv Sci Technol 2023;87:104753.

[48] Antoniou V , Mourelatou EA, Galatou E, Avgoustakis K, Hatziantoniou S. Gene therapy with chitosan nanoparticles: modern formulation strategies for enhancing cancer cell transfection. Pharmaceutics 2024;16:868. 39065565 10.3390/pharmaceutics16070868 PMC11280172

[49] Ashrafizadeh M , Delfi M, Hashemi F, Zabolian A, Saleki H, Bagherian M, Azami N, Farahani MV, Sharifzadeh SO, Hamzehlou S, Hushmandi K, Makvandi P, Zarrabi A, Hamblin MR, Varma RS. Biomedical application of chitosan-based nanoscale delivery systems: potential usefulness in siRNA delivery for cancer therapy. Carbohydr Polym 2021;260:117809. 33712155 10.1016/j.carbpol.2021.117809

[50] Liu C , Tang C, Yin C. Co-delivery of doxorubicin and siRNA by all-trans retinoic acid conjugated chitosan-based nanocarriers for multiple synergistic antitumor efficacy. Carbohydr Polym 2022;283:119097. 35153031 10.1016/j.carbpol.2022.119097

[51] Sun H , Zhang L, Zhao N, Xin H. Cu^2+^-citrate-chitosan complex nanoparticles for the chemodynamic therapy of lung cancer. ACS Omega 2024;9:8425–33. 38405439 10.1021/acsomega.3c09619 PMC10883013

[52] Butsyk A , Varava Y, Moskalenko R, Husak Y, Piddubnyi A, Denysenko A, Korniienko V, Ramanaviciute A, Banasiuk R, Pogorielov M, Ramanavicius A, Korniienko V. Copper nanoparticle loaded electrospun patches for infected wound treatment: from development to in-vivo application. Polymers 2024;16:2733. 39408444 10.3390/polym16192733 PMC11479054

[53] Cao J , Zhu C, Cao Z, Ke X. CPPs-modified chitosan as permeability-enhancing chemotherapeutic combined with gene therapy nanosystem by thermosensitive hydrogel for the treatment of osteosarcoma. Int J Biol Macromol 2024;267:130915. 38561118 10.1016/j.ijbiomac.2024.130915

[54] Wu K , Yu B, Li D, Tian Y, Liu Y, Jiang J. Recent advances in nanoplatforms for the treatment of osteosarcoma. Front Oncol 2022;12:805978. 35242707 10.3389/fonc.2022.805978 PMC8885548

[55] Chen M , Li M, Wei Y, Xue C, Chen M, Fei Y, Tan L, Luo Z, Cai K, Hu Y. ROS-activatable biomimetic interface mediates in-situ bioenergetic remodeling of osteogenic cells for osteoporotic bone repair. Biomaterials 2022;291:121878. 36335716 10.1016/j.biomaterials.2022.121878

[56] Mahmudi H , Adili-Aghdam MA, Shahpouri M, Jaymand M, Amoozgar Z, Jahanban-Esfahlan R. Tumor microenvironment penetrating chitosan nanoparticles for elimination of cancer relapse and minimal residual disease. *Front Oncol* 2022;12:1054029. 36531004 10.3389/fonc.2022.1054029 PMC9751059

[57] Ismail EA , Omolo CA, Gafar MA, Khan R, Nyandoro VO, Salifu EY, Govender T. Multi-functional pH-responsive and biomimetic chitosan-based nanoplexes for targeted delivery of ciprofloxacin against bacterial sepsis. Int J Biol Macromol 2024;262:130046. 38336334 10.1016/j.ijbiomac.2024.130046

[58] Bhattacharya S , Prajapati BG, Singh S. A critical review on the dissemination of PH and stimuli-responsive polymeric nanoparticular systems to improve drug delivery in cancer therapy. Crit Rev Oncol Hematol 2023;185:103961. 36921781 10.1016/j.critrevonc.2023.103961

[59] Herdiana Y , Wathoni N, Gozali D, Shamsuddin S, Muchtaridi M. Chitosan-based nano-smart drug delivery system in breast cancer therapy. Pharmaceutics 2023;15:879. 36986740 10.3390/pharmaceutics15030879 PMC10051865

[60] Bordone M , Bettencourt A. Management of bone diseases: looking at scaffold-based strategies for drug delivery. Drug Deliv Transl Res 2023;13:79–104. 35816230 10.1007/s13346-022-01191-w

[61] Sun C , Li S, Ding J. Biomaterials-boosted immunotherapy for osteosarcoma. Adv Healthc Mater 2024;13:2400864. 10.1002/adhm.202400864 38771618

[62] Pan C , Ji Z, Wang Q, Zhang Z, Wang Z, Li C, Lu S, Ge P. Cuproptosis: mechanisms, biological significance, and advances in disease treatment—a systematic review, CNS Neurosci Ther 2024;30:e70039. 39267265 10.1111/cns.70039 PMC11392831

[63] Tharakan S , Raja I, Pietraru A, Sarecha E, Gresita A, Petcu E, Ilyas A, Hadjiargyrou M. The use of hydrogels for the treatment of bone osteosarcoma via localized drug-delivery and tissue regeneration: a narrative review. Gels 2023;9:274. 37102886 10.3390/gels9040274 PMC10137556

[64] Mahar R , Chakraborty A, Nainwal N, Bahuguna R, Sajwan M, Jakhmola V. Application of PLGA as a biodegradable and biocompatible polymer for pulmonary delivery of drugs. AAPS PharmSciTech 2023;24:39. 36653547 10.1208/s12249-023-02502-1

[65] Al Bostami RD , Abuwatfa WH, Husseini GA. Recent advances in nanoparticle-based co-delivery systems for cancer therapy. Nanomaterials 2022;12:2672. 35957103 10.3390/nano12152672 PMC9370272

[66] El-Hammadi MM , Arias JL. Recent advances in the surface functionalization of PLGA-based nanomedicines. Nanomaterials 2022;12:354. 35159698 10.3390/nano12030354 PMC8840194

[67] Dinakaran D , Azad AK, Wilson BC. Chapter 13- Photodynamic and photothermal therapy using PLGA nanoparticles. In: Kesharwani P (ed). Poly(Lactic-Co-Glycolic Acid) (PLGA) Nanoparticles for Drug Delivery. Amsterdam, Netherlands: Elsevier, 2023;357–91.

[68] Pallavi P , Sharmiladevi P, Haribabu V, Girigoswami K, Girigoswami A. A nano approach to formulate photosensitizers for photodynamic therapy. Curr Nanosci 2022;18:675–89.

[69] Alvi M , Yaqoob A, Rehman K, Shoaib SM, Akash MSH. PLGA-based nanoparticles for the treatment of cancer: current strategies and perspectives. AAPS Open 2022;8:12.

[70] María Paulina R , Myriam Alexandra G. Chapter 2-Drug-delivery based on encapsulation for photodynamic therapy and photothermal therapy. In: Ashutosh S (ed). Biomaterials in Microencapsulation. Rijeka: IntechOpen, 2024.

[71] Luo Y , Sun M, Tan L, Li T, Min L. Nano-based drug delivery systems: potential developments in the therapy of metastatic osteosarcoma—a narrative review. Pharmaceutics 2023;15:2717. 38140058 10.3390/pharmaceutics15122717 PMC10747574

[72] Paltanea G , Manescu V, Antoniac I, Antoniac A, Nemoianu IV, Robu A, Dura H. A review of biomimetic and biodegradable magnetic scaffolds for bone tissue engineering and oncology. Int J Mol Sci 2023;24:4312. 36901743 10.3390/ijms24054312 PMC10001544

[73] Shah SR , Modi CD, Singh S, Mori DD, Soniwala MM, Prajapati BG. Recent advances in additive manufacturing of polycaprolactone-based scaffolds for tissue engineering applications: a comprehensive review. Regen Eng Transl Med 2025;11:112–31.

[74] El Gohary NA , Mahmoud A, Ashraf Nazmy M, Zaabalawi R, El Zahar L, Khalil ISM, Mitwally ME. Magnetic polycaprolactone microspheres: drug encapsulation and control. Int J Polym Mater Polym Biomater 2024;73:143–53.

[75] Yue L. Synthesis and application of partial block polymers in drug delivery. Highl Sci Eng Technol 2024;91:322–7.

[76] Khan M , Yamasta A, Parvin M, Ferdaus J, Ahmed H, Arbab AS. Experimental study of processing of PCL (polycaprolactone)-peptides nanoparticles and its biodistribution analysis for drug delivery system. Micro Nano Syst Lett 2024;12:18.

[77] Jaswal R , Kumar D, Kaliannagounder VK, Rezk AI, Kandel R, Park CH, Min KH. Osteopromotive PDA-modified gold nanoparticles-incorporated bioinspired polycaprolactone-based nanofibers for bone cancer therapy and robust bone regeneration. Mater Today Nano 2024;25:100453.

[78] Huang B , Yin Z, Zhou F, Su J. Functional anti-bone tumor biomaterial scaffold: construction and application. J Mater Chem B 2023;11:8565–85. 37415547 10.1039/d3tb00925d

[79] Tian J , Paterson TE, Zhang J, Li Y, Ouyang H, Asencio IO, Hatton PV, Zhao Y, Li Z. Enhanced antibacterial ability of electrospun PCL scaffolds incorporating ZnO nanowires. Int J Mol Sci 2023;24:14420. 37833866 10.3390/ijms241914420 PMC10572921

[80] Florensa M , Llenas M, Medina-Gutiérrez E, Sandoval S, Tobías-Rossell G. Key parameters for the rational design, synthesis, and functionalization of biocompatible mesoporous silica nanoparticles. Pharmaceutics 2022;14:2703. 36559195 10.3390/pharmaceutics14122703 PMC9788600

[81] Ahmed H , Gomte SS, Prathyusha E, A P, Agrawal M, Alexander A. Biomedical applications of mesoporous silica nanoparticles as a drug delivery carrier. J Drug Deliv Sci Technol 2022;76:103729.

[82] Chao B , Jiao J, Yang L, Wang Y, Jiang W, Yu T, Wang L, Liu H, Zhang H, Wang Z, Wu M. Application of advanced biomaterials in photothermal therapy for malignant bone tumors. Biomater Res 2023;27:116. 37968707 10.1186/s40824-023-00453-z PMC10652612

[83] Vallet-Regí M. Our contributions to applications of mesoporous silica nanoparticles. Acta Biomater 2022;137:44–52. 34653693 10.1016/j.actbio.2021.10.011

[84] He L , Javid Anbardan Z, Habibovic P, van Rijt S. Doxorubicin- and selenium-incorporated mesoporous silica nanoparticles as a combination therapy for osteosarcoma. ACS Appl Nano Mater 2024;7:25400–11. 39606122 10.1021/acsanm.4c04294 PMC11590048

[85] Yuan P , Min Y, Zhao Z. Multifunctional nanoparticles for the treatment and diagnosis of osteosarcoma. Biomater Adv 2023;151:213466. 37229927 10.1016/j.bioadv.2023.213466

[86] Vahidi M , Rizkalla AS, Mequanint K. Extracellular matrix-surrogate advanced functional composite biomaterials for tissue repair and regeneration. Adv Healthc Mater 2024;13:2401218. 39036851 10.1002/adhm.202401218 PMC12344620

[87] Kavitha Sri A , Arthi C, Neya NR, Hikku GS. Nano-hydroxyapatite/collagen composite as scaffold material for bone regeneration. Biomed Mater 2023;18:032002 10.1088/1748-605X/acc99e 37001544

[88] Zakeri N , Rezaie HR, Javadpour J, Kharaziha M. Cisplatin loaded polycaprolactone—zeolite nanocomposite scaffolds for bone cancer treatment. J Sci: Adv Mater Dev 2022;7:100377.

[89] De Lama-Odría M , del Valle LJ, Puiggalí J. Hydroxyapatite biobased materials for treatment and diagnosis of cancer. Int J Mol Sci 2022;23:11352. 36232652 10.3390/ijms231911352 PMC9569977

[90] Han D , Wang W, Gong J, Ma Y, Li Y. Collagen-hydroxyapatite based scaffolds for bone trauma and regeneration: recent trends and future perspectives. Nanomedicine (Lond) 2024;19:1689–709. 39163266 10.1080/17435889.2024.2375958 PMC11389751

[91] Radulescu D-E , Neacsu IA, Grumezescu A-M, Andronescu E. Novel trends into the development of natural hydroxyapatite-based polymeric composites for bone tissue engineering. Polymers 2022;14:899. 35267722 10.3390/polym14050899 PMC8912671

[92] Ghosh R , Das S, Mallick SP, Beyene Z. A review on the antimicrobial and antibiofilm activity of doped hydroxyapatite and its composites for biomedical applications. Mater Today Commun 2022;31:103311.

[93] Wu Y , Cheng M, Jiang Y, Zhang X, Li J, Zhu Y, Yao Q. Calcium-based biomaterials: unveiling features and expanding applications in osteosarcoma treatment. Bioact Mater 2024;32:385–99. 37920827 10.1016/j.bioactmat.2023.10.008 PMC10618625

[94] Shao R , Wang Y, Li L, Dong Y, Zhao J, Liang W. Bone tumors effective therapy through functionalized hydrogels: current developments and future expectations. Drug Deliv 2022;29:1631–47. 35612368 10.1080/10717544.2022.2075983 PMC9154780

[95] Kang N-W , Lee J-Y, Kim D-D. Hydroxyapatite-binding albumin nanoclusters for enhancing bone tumor chemotherapy. J Control Release 2022;342:111–21. 34990700 10.1016/j.jconrel.2021.12.039

[96] Ahmed MK , Mansour SF, Al-Wafi R, Abdel-Fattah E. Nanofibers scaffolds of co-doped Bi/Sr-hydroxyapatite encapsulated into polycaprolactone for biomedical applications. J Mater Res Technol 2021;13:2297–309.

[97] Klotz BJ , Gawlitta D, Rosenberg A, Malda J, Melchels FPW. Gelatin-methacryloyl hydrogels: towards biofabrication-based tissue repair. Trends Biotechnol 2016;34:394–407. 26867787 10.1016/j.tibtech.2016.01.002 PMC5937681

[98] Huang K , Liu W, Wei W, Zhao Y, Zhuang P, Wang X, Wang Y, Hu Y, Dai H. Photothermal hydrogel encapsulating intelligently bacteria-capturing bio-MOF for infectious wound healing. ACS Nano 2022;16:19491–508. 36321923 10.1021/acsnano.2c09593

[99] Kulkarni NS , Chauhan G, Goyal M, Sarvepalli S, Gupta V. Development of gelatin methacrylate (GelMa) hydrogels for versatile intracavitary applications. Biomater Sci 2022;10:4492–507. 35786706 10.1039/d2bm00022a

[100] Singh A , Dalal N, Tayalia P. An interplay of matrix stiffness, dimensionality and adhesivity on cellular behavior. Biomed Mater 2023;18:025010. 10.1088/1748-605X/acb7c0 36720169

[101] Rodrigues J , Sarmento B, Pereira CL. Osteosarcoma tumor microenvironment: the key for the successful development of biologically relevant 3D in vitro models. In Vitro Model 2022;1:5–27. 39872973 10.1007/s44164-022-00008-x PMC11756501

[102] Jiao W , Shan J, Gong X, Sun Y, Sang L, Ding X, Zhou H, Yu M. GelMA hydrogel: a game-changer in 3D tumor modeling. Mater Today Chem 2024;38:102111.

[103] Das S , Jegadeesan JT, Basu B. Gelatin methacryloyl (GelMA)-based biomaterial inks: process science for 3D/4D printing and current status. Biomacromolecules 2024;25:2156–221. 38507816 10.1021/acs.biomac.3c01271

[104] Gupta AK , Choudhari A, Kumar A, Kumar A, Gupta A, Faisal S, Kumar A. Composites for drug-eluting devices: emerging biomedical applications. In: Kumar A, Kumar A, Kumar A (eds). Applications of Biotribology in Biomedical Systems. Cham: Springer Nature Switzerland, 2024, 251–311.

[105] Shi P , Cheng Z, Zhao K, Chen Y, Zhang A, Gan W, Zhang Y. Active targeting schemes for nano-drug delivery systems in osteosarcoma therapeutics. J Nanobiotechnology 2023;21:103. 36944946 10.1186/s12951-023-01826-1 PMC10031984

[106] Kargozar S , Mollazadeh S, Kermani F, Webster TJ, Nazarnezhad S, Hamzehlou S, Baino F. Hydroxyapatite nanoparticles for improved cancer theranostics. J Funct Biomater 2022;13:100. 35893468 10.3390/jfb13030100 PMC9326646

[107] Yu K , Zhou H, Xu Y, Cao Y, Zheng Y, Liang B. Engineering a triple-functional magnetic gel driving mutually-synergistic mild hyperthermia-starvation therapy for osteosarcoma treatment and augmented bone regeneration. J Nanobiotechnology 2023;21:201. 37365598 10.1186/s12951-023-01955-7 PMC10291780

[108] Liu X , Lu S, Wang T, Wang X, Yang K, Yang H. Advances and prospects of 3D printed antibacterial bone implants: a systematic review. J Mater Sci Technol 2024;200:227–42.

[109] Liang W , Dong Y, Shao R, Zhang S, Wu X, Huang X, Sun B, Zeng B, Zhao J. Application of nanoparticles in drug delivery for the treatment of osteosarcoma: focussing on the liposomes. J Drug Target 2022;30:463–75. 34962448 10.1080/1061186X.2021.2023160

[110] Burdușel A-C , Andronescu E. Lipid nanoparticles and liposomes for bone diseases treatment. Biomedicines 2022;10:3158. 36551914 10.3390/biomedicines10123158 PMC9775639

[111] Hu B , Zhang Y, Zhang G, Li Z, Jing Y, Yao J, Sun S. Research progress of bone-targeted drug delivery system on metastatic bone tumors. J Control Release 2022;350:377–88. 36007681 10.1016/j.jconrel.2022.08.034

[112] Chen Z , Wang X, Zhao N, Chen H, Guo G. Advancements in pH-responsive nanocarriers: enhancing drug delivery for tumor therapy. Expert Opin Drug Deliv 2023;20:1623–42. 38059646 10.1080/17425247.2023.2292678

[113] Amin M , Lammers T, ten Hagen TLM. Temperature-sensitive polymers to promote heat-triggered drug release from liposomes: towards bypassing EPR. Adv Drug Deliv Rev 2022;189:114503. 35998827 10.1016/j.addr.2022.114503

[114] Wang M , Xu D, Xu C, Li M, Du C, Liu Y. Ultrasound-activated multifunctional bioactive calcium phosphate composites for enhanced osteosarcoma treatment. Coatings 2024;14:1267.

[115] Celik B , Cicek K, Leal AF, Tomatsu S. Regulation of molecular targets in osteosarcoma treatment. Int J Mol Sci 2022;23:12583. 36293439 10.3390/ijms232012583 PMC9604206

[116] Li M , Lin Z-I, Yang J, Huang H, Liu G-L, Liu Q, Zhang X, Zhang Y, Xu Z, Lin H, Chai Y, Chen X, Ko B-T, Liu J, Chen C-K, Yang C. Biodegradable carbon dioxide-derived non-viral gene vectors for osteosarcoma gene therapy. Adv Healthc Mater 2023;12:2201306. 10.1002/adhm.202201306 36308025

[117] Chen W , Li H, Shi D, Liu Z, Yuan W. Microneedles as a delivery system for gene therapy. Front Pharmacol 2016;7:137. 27303298 10.3389/fphar.2016.00137 PMC4880556

[118] Pei P , Yang F, Liu J, Hu H, Du X, Hanagata N, Zhao S, Zhu Y. Composite-dissolving microneedle patches for chemotherapy and photothermal therapy in superficial tumor treatment. Biomater Sci 2018;6:1414–23. 29595852 10.1039/c8bm00005k

[119] Xu K , Fei W, Huo Z, Wang S, Li Y, Yang G, Hong Y. PDCD10 promotes proliferation, migration, and invasion of osteosarcoma by inhibiting apoptosis and activating EMT pathway. Cancer Med 2023;12:1673–84. 35848121 10.1002/cam4.5025 PMC9883585

[120] Damiri F , Fatimi A, Liu Y, Musuc AM, Fajardo AR, Gowda BHJ, Vora LK, Shavandi A, Okoro OV. Recent advances in 3D bioprinted polysaccharide hydrogels for biomedical applications: a comprehensive review. Carbohydr Polym 2025;348:122845. 39567171 10.1016/j.carbpol.2024.122845

[121] Ta HT , Dass CR, Larson I, Choong PF, Dunstan DE. A chitosan hydrogel delivery system for osteosarcoma gene therapy with pigment epithelium-derived factor combined with chemotherapy. Biomaterials 2009;30:4815–23. 19505719 10.1016/j.biomaterials.2009.05.035

[122] Dhuri A , Kanp T, Rode K, M B, Gupta U, Guru SK, Singh PK. Current advances in non-viral nanoparticle-based gene therapy for effective management of cancer. J Drug Deliv Sci Technol 2024;100:106083.

[123] Wang C , Zhang Y, Kong W, Rong X, Zhong Z, Jiang L, Chen S, Li C, Zhang F, Jiang J. Delivery of miRNAs using nanoparticles for the treatment of osteosarcoma. Int J Nanomedicine 2024;19:8641–60. 39188861 10.2147/IJN.S471900 PMC11346496

[124] Singh P , Singh M, Singh B, Sharma K, Kumar N, Singh D, Klair HS, Mastana S. Implications of siRNA therapy in bone health: silencing communicates. Biomedicines 2024;12:90. 38255196 10.3390/biomedicines12010090 PMC10813040

[125] Giordano F , Lenna S, Baudo G, Rampado R, Massaro M, De Rosa E, Ewing A, Kurenbekova L, Agostini M, Yustein JT, Taraballi F. Tyrosine kinase inhibitor-loaded biomimetic nanoparticles as a treatment for osteosarcoma. Cancer Nano 2022;13:40.

[126] Adachi T , Tahara Y, Yamamoto K, Yamamoto T, Kanamura N, Akiyoshi K, Mazda O. Cholesterol-bearing polysaccharide-based nanogels for development of novel immunotherapy and regenerative medicine. Gels 2024;10:206. 38534624 10.3390/gels10030206 PMC10970560

[127] Li K , Zhang Z, Mei Y, Li M, Yang Q, Wu Q, Yang H, He L, Liu S. Targeting the innate immune system with nanoparticles for cancer immunotherapy. J Mater Chem B 2022;10:1709–33. 35179545 10.1039/d1tb02818a

[128] Zhang Z , Yao S, Hu Y, Zhao X, Lee RJ. Application of lipid-based nanoparticles in cancer immunotherapy. Front Immunol 2022;13:967505. 36003395 10.3389/fimmu.2022.967505 PMC9393708

[129] Zang C , Tian Y, Tang Y, Tang M, Yang D, Chen F, Ghaffarlou M, Tu Y, Ashrafizadeh M, Li Y. Hydrogel-based platforms for site-specific doxorubicin release in cancer therapy. J Transl Med 2024;22:879. 39350207 10.1186/s12967-024-05490-3 PMC11440768

[130] Cheng Z , Fobian S-F, Gurrieri E, Amin M, D'Agostino VG, Falahati M, Zalba S, Debets R, Garrido MJ, Saeed M, Seynhaeve ALB, Balcioglu HE, Ten Hagen TLM. Lipid-based nanosystems: the next generation of cancer immune therapy. J Hematol Oncol 2024;17:53. 39030582 10.1186/s13045-024-01574-1 PMC11265205

[131] Quadros M , Momin M, Verma G. Design strategies and evolving role of biomaterial assisted treatment of osteosarcoma. Mater Sci Eng C Mater Biol Appl 2021;121:111875. 33579498 10.1016/j.msec.2021.111875

[132] Wei H , Cui J, Lin K, Xie J, Wang X. Recent advances in smart stimuli-responsive biomaterials for bone therapeutics and regeneration. Bone Res 2022;10:17. 35197462 10.1038/s41413-021-00180-y PMC8866424

[133] Rahim MA , Jan N, Khan S, Shah H, Madni A, Khan A, Jabar A, Khan S, Elhissi A, Hussain Z, Aziz HC, Sohail M, Khan M, Thu HE. Recent advancements in stimuli responsive drug delivery platforms for active and passive cancer targeting. Cancers (Basel) 2021;13:670. 33562376 10.3390/cancers13040670 PMC7914759

[134] Zhu J , Gao R, Wang Z, Cheng Z, Xu Z, Liu Z, Wu Y, Wang M, Zhang Y. Sustained and targeted delivery of self-assembled doxorubicin nonapeptides using pH-responsive hydrogels for osteosarcoma chemotherapy. Pharmaceutics 2023;15:668. 36839990 10.3390/pharmaceutics15020668 PMC9961168

[135] Ren X , Liu H, Wu X, Weng W, Wang X, Su J. Reactive oxygen species (ROS)-responsive biomaterials for the treatment of bone-related diseases. Front Bioeng Biotechnol 2021;9:820468. 35087811 10.3389/fbioe.2021.820468 PMC8787194

[136] Lin H , Jin X, Cao Y, Ruan R, Liu C, Huang S, Xu J, Ding J, Yang H, Zhang J. Self-adaptive hydrogel with Cascade microenvironments-responsiveness to inhibit osteosarcoma progression and augment bone reconstruction. Adv Funct Mater 2025;35:2421470.

[137] Qian Y , Lu S, Meng J, Chen W, Li J. Thermo-responsive hydrogels coupled with photothermal agents for biomedical applications. Macromol Biosci 2023;23:e2300214. 37526220 10.1002/mabi.202300214

[138] Shan H , Li K, Zhao D, Chi C, Tan Q, Wang X, Yu J, Piao M. Locally controlled release of methotrexate and alendronate by thermo-sensitive hydrogels for synergistic inhibition of osteosarcoma progression. Front Pharmacol 2020;11:573. 32508628 10.3389/fphar.2020.00573 PMC7248331

[139] Katiyar D. Applications of intelligent biomaterials in cancer immunotherapy: a review. Mater Today: Proc 2023. Doi: 10.1016/j.matpr.2023.05.460.

[140] Xu L , Cao Y, Xu Y, Li R, Xu X. Redox-responsive polymeric nanoparticle for nucleic acid delivery and cancer therapy: progress, opportunities, and challenges. Macromol Biosci 2024;24:e2300238. 37573033 10.1002/mabi.202300238 PMC13420779

[141] Chen S , Wang Y, Zhang X, Ma J, Wang M. Double-crosslinked bifunctional hydrogels with encapsulated anti-cancer drug for bone tumor cell ablation and bone tissue regeneration. Colloids Surf B Biointerfaces 2022;213:112364. 35219965 10.1016/j.colsurfb.2022.112364

[142] Hanaei SB , Murugesan RC, Souza LP, Cadiz-Miranda JI, Jeys L, Wall IB, Martin RA. Multifunctional gallium doped bioactive glasses: a targeted delivery for antineoplastic agents and tissue repair against osteosarcoma. Biomed Mater 2024;19:065008. 10.1088/1748-605X/ad76f1 39226916

[143] Amini Z , Rudsary SS, Shahraeini SS, Dizaji BF, Goleij P, Bakhtiari A, Irani M, Sharifianjazi F. Magnetic bioactive glasses/cisplatin loaded-chitosan (CS)-grafted- poly (ε-caprolactone) nanofibers against bone cancer treatment. Carbohydr Polym 2021;258:117680. 33593554 10.1016/j.carbpol.2021.117680

[144] Lv Y , Su L, Zhao Z, Zhao J, Su H, Zhang Z, Wang Y. Chitosan microspheres loaded with curcumin and gallic acid: modified synthesis, sustainable slow release, and enhanced biological property. Curr Microbiol 2023;80:240. 37296240 10.1007/s00284-023-03352-7

[145] Lončarević A , Clara-Trujillo S, Martínez-Férriz A, Blanco-Gómez M, Gallego-Ferrer G, Rogina A. Chitosan-copper microparticles as doxorubicin microcarriers for bone tumor therapy. Int J Pharm 2024;659:124245. 38772497 10.1016/j.ijpharm.2024.124245

[146] Marshall SK , Taweesap M, Saelim B, Pachana V, Benlateh N, Sangangam S, Bumrungsin A, Kholo-Asae H, Wongtechanon I. Cytotoxicity enhancement in osteosarcoma with multifunctional I-131 radiotherapeutic nanoparticles: in vitro three-dimensional spheroid model and release kinetics modeling. Molecules 2024;29:630. 38338373 10.3390/molecules29030630 PMC10856476

[147] Lin Y , Wang B. pH-responsive paclitaxel prodrug encapsulated in a polypeptide-chitosan polymer delivery system for osteosarcoma treatment. Carbohydr Res 2025;551:109414. 39923605 10.1016/j.carres.2025.109414

[148] Abdel-Rahman RM , Abdel-Mohsen AM, Frankova J, Piana F, Kalina L, Gajdosova V, Kapralkova L, Thottappali MA, Jancar J. Self-assembled hydrogel membranes with structurally tunable mechanical and biological properties. Biomacromolecules 2024;25:3449–63. 38739908 10.1021/acs.biomac.4c00082 PMC11170955

[149] Bulygina IN , Karshieva SS, Permyakova ES, Korol AA, Kolesnikov EA, Choudhary R, Senatov FS, Koudan EV. In vitro evaluation of doxorubicin release from diopside particles on MG-63 and HF spheroids as a 3D model of tumor and healthy tissues. Toxicol In Vitro 2024;98:105830. 38641231 10.1016/j.tiv.2024.105830

[150] Stepanova M , Dobrodumov A, Averianov I, Gofman I, Nashchekina J, Guryanov I, Klyukin I, Zhdanov A, Korzhikova-Vlakh E, Zhizhin K. Design, fabrication and characterization of biodegradable composites containing *Closo*-Borates as potential materials for boron neutron capture therapy. Polymers (Basel) 2022;14:3864. 36146012 10.3390/polym14183864 PMC9506383

[151] Sundarakrishnan A. Extremely rapid gelling curcumin silk-tyrosine crosslinked hydrogels. Gels 2025;11:288. 40277724 10.3390/gels11040288 PMC12027036

[152] Khaled Wassif R , Daihom BA, Maniruzzaman M. FRESH 3D printing of zoledronic acid-loaded chitosan/alginate/hydroxyapatite composite thermosensitive hydrogel for promoting bone regeneration. Int J Pharm 2024;667:124898. 39500473 10.1016/j.ijpharm.2024.124898

[153] Mukundan LM , Nirmal SR, Kumar N, Dhara S, Chattopadhyay S. Engineered nanostructures within sol-gel bioactive glass for enhanced bioactivity and modulated drug delivery. J Mater Chem B 2022;10:10112–27. 36468610 10.1039/d2tb01692c

[154] George LH , Arakkal A, Sreedharan P, Sailaja GS. Injectable polyelectrolyte complex-nascent HAP biodegradable antibiotic delivery system for the treatment of osteomyelitis. Biomed Mater 2021;17:015011. 10.1088/1748-605X/ac37c5 34753122

[155] Xu D , Wan Y, Xie Z, Du C, Wang Y. Hierarchically structured hydroxyapatite particles facilitate the enhanced integration and selective anti-tumor effects of amphiphilic prodrug for osteosarcoma therapy. Adv Healthc Mater 2023;12:e2202668. 36857811 10.1002/adhm.202202668

[156] Bhattacharjee A , Jo Y, Bose S. In vivo and In vitro properties evaluation of curcumin loaded MgO doped 3D printed TCP scaffolds. J Mater Chem B 2023;11:4725–39. 37171110 10.1039/d2tb02547g PMC10314738

[157] Jo Y , Kushram P, Bose S. Curcumin and vitamin D3 release from calcium phosphate enhances bone regeneration. Biomater Sci 2025;13:1568–77. 39960074 10.1039/d4bm01188k PMC13182675

[158] Martinez T , Sarda S, Dupret-Bories A, Charvillat C, Projetti F, Drouet C. Toward a doxorubicin-loaded bioinspired bone cement for the localized treatment of osteosarcoma. Future Oncol 2021;17:3511–28. 34213375 10.2217/fon-2021-0128

[159] Kovrlija I , Pańczyszyn E, Demir O, Laizane M, Corazzari M, Locs J, Loca D. Doxorubicin loaded octacalcium phosphate particles as controlled release drug delivery systems: physico-chemical characterization, in vitro drug release and evaluation of cell death pathway. Int J Pharm 2024;653:123932. 38387818 10.1016/j.ijpharm.2024.123932

[160] Liu Y , Raina DB, Sebastian S, Nagesh H, Isaksson H, Engellau J, Lidgren L, Tägil M. Sustained and controlled delivery of doxorubicin from an in-situ setting biphasic hydroxyapatite carrier for local treatment of a highly proliferative human osteosarcoma. Acta Biomater 2021;131:555–71. 34271171 10.1016/j.actbio.2021.07.016

[161] Liu J , Lin S, Dang J, Wang S, Cheng W, Ran Z, Zhu H, Deng H, Xiong C, Xu W, Huang Z, Xu P, Xu H. Anticancer and bone-enhanced nano-hydroxyapatite/gelatin/polylactic acid fibrous membrane with dual drug delivery and sequential release for osteosarcoma. Int J Biol Macromol 2023;240:124406. 37060976 10.1016/j.ijbiomac.2023.124406

[162] Ganesan V , Meiyazhagan G, Devaraj M, Kandasamy S, Manogaran P, Suresh Kumar G, Raji G, Kattimani VS, Easwaradas Kreedapathy G. Repurposing the antibacterial activity of etoposide—a chemotherapeutic drug in combination with eggshell-derived hydroxyapatite. ACS Biomater Sci Eng 2022;8:682–93. 35050575 10.1021/acsbiomaterials.1c01481

[163] Unnikrishnan V , Venugopal A, Sivadasan SB, Boniface Fernandez F, Arumugam S, P RH, Parayanthala Valappil M. Cellular and sub-chronic toxicity of hydroxyapatite porous beads loaded with antibiotic in rabbits, indented for chronic osteomyelitis. Int J Pharm 2022;616:121535. 35124118 10.1016/j.ijpharm.2022.121535

[164] Tan W , Gao C, Feng P, Liu Q, Liu C, Wang Z, Deng Y, Shuai C. Dual-functional scaffolds of poly(L-lactic acid)/nanohydroxyapatite encapsulated with metformin: simultaneous enhancement of bone repair and bone tumor inhibition. Mater Sci Eng C Mater Biol Appl 2021;120:111592. 33545810 10.1016/j.msec.2020.111592

[165] Wu H , Wang R, Li S, Chen S, Liu S, Li X, Yang X, Zeng Q, Zhou Y, Zhu X, Zhang K, Tu C, Zhang X. Aspect ratio-dependent dual-regulation of the tumor immune microenvironment against osteosarcoma by hydroxyapatite nanoparticles. Acta Biomater 2023;170:427–41. 37634831 10.1016/j.actbio.2023.08.046

[166] Toulou C , Chaudhari VS, Bose S. Extrusion 3D-printed tricalcium phosphate-polycaprolactone biocomposites for quercetin-KCl delivery in bone tissue engineering. J Biomed Mater Res A 2024;112:1472–83. 38477071 10.1002/jbm.a.37692 PMC11239310

[167] Szurkowska K , Kazimierczak P, Kolmas J. Mg, Si-Co-substituted hydroxyapatite/alginate composite beads loaded with raloxifene for potential use in bone tissue regeneration. Int J Mol Sci 2021;22:2933. 33805785 10.3390/ijms22062933 PMC7999305

[168] Kameda Y , Aizawa M, Sato T, Honda M. Zoledronic acid-loaded β-TCP inhibits tumor proliferation and osteoclast activation: development of a functional bone substitute for an efficient osteosarcoma treatment. Int J Mol Sci 2021;22:1889. 33672879 10.3390/ijms22041889 PMC7918630

[169] Zhong J , Wen W, Wang J, Zhang M, Jia Y, Ma X, Su YX, Wang Y, Lan X. Bone-targeted dual functional lipid-coated drug delivery system for osteosarcoma therapy. Pharm Res 2023;40:231–43. 36380167 10.1007/s11095-022-03430-8 PMC9666974

[170] Chotchindakun K , Pekkoh J, Ruangsuriya J, Zheng K, Unalan I, Boccaccini AR. Fabrication and characterization of cinnamaldehyde-loaded mesoporous bioactive glass nanoparticles/PHBV-based microspheres for preventing bacterial infection and promoting bone tissue regeneration. Polymers (Basel) 2021;13:1794. 34072334 10.3390/polym13111794 PMC8198921

[171] Ghafouri F , Dehghanian Reyhan V, Sadeghi M, Miraei-Ashtiani SR, Kastelic JP, Barkema HW, Shirali M. Competing endogenous RNAs (ceRNAs) and application of their regulatory networks in complex traits and diseases of ruminants. Ruminants 2024;4:165–81.

[172] Gao J , Huang J, Shi R, Wei J, Lei X, Dou Y, Li Y, Zuo Y, Li J. Loading and releasing behavior of selenium and doxorubicin hydrochloride in hydroxyapatite with different morphologies. ACS Omega 2021;6:8365–75. 33817497 10.1021/acsomega.1c00092 PMC8015115

[173] He L , Habibovic P, van Rijt S. Selenium-incorporated mesoporous silica nanoparticles for osteosarcoma therapy. Biomater Sci 2023;11:3828–39. 37074160 10.1039/d2bm02102a PMC10227887

[174] Tan B , Wu Y, Wu Y, Shi K, Han R, Li Y, Qian Z, Liao J. Curcumin-microsphere/IR820 hybrid bifunctional hydrogels for in situ osteosarcoma chemo-co-thermal therapy and bone reconstruction. ACS Appl Mater Interfaces 2021;13:31542–53. 34191477 10.1021/acsami.1c08775

[175] Gupta S , Qayoom I, Gupta P, Gupta A, Singh P, Singh S, Kumar A. Exosome-functionalized, drug-laden bone substitute along with an antioxidant herbal membrane for bone and periosteum regeneration in bone sarcoma. ACS Appl Mater Interfaces 2023;15:8824–39. 36749176 10.1021/acsami.2c18308

[176] Amiryaghoubi N , Abdolahinia ED, Nakhlband A, Aslzad S, Fathi M, Barar J, Omidi Y. Smart chitosan-folate hybrid magnetic nanoparticles for targeted delivery of doxorubicin to osteosarcoma cells. Colloids Surf B Biointerfaces 2022;220:112911. 36274396 10.1016/j.colsurfb.2022.112911

[177] Vo TMT , Mondal S, Nguyen VT, Park S, Choi J, Bui NT, Oh J. Rice starch coated iron oxide nanoparticles: a theranostic probe for photoacoustic imaging-guided photothermal cancer therapy. Int J Biol Macromol 2021;183:55–67. 33857520 10.1016/j.ijbiomac.2021.04.053

[178] Caldas M , Barbosa AI, Bhattacharya M, Reis RL, Correlo VM. Natural melanin nanoparticles (MNPs) extracted from *Sepia officinalis*: a cost-effective, chemo-photothermal, synergistic nanoplatform for osteosarcoma treatment. Colloids Surf B Biointerfaces 2024;239:113937. 38749166 10.1016/j.colsurfb.2024.113937

[179] Baniahmad F , Yousefi S, Rabiee M, Sara Shafiei S, Faghihi S. Alendronate sodium intercalation in layered double hydroxide/poly (ε-caprolactone): application in osteoporosis treatment. Iran J Biotechnol 2021;19:e2490. 34179186 10.30498/IJB.2021.2490 PMC8217540

[180] Thepsri N , Kaewsrichan J. Triple-layered scaffold containing cisplatin and curcumin applied for cancerous bone regeneration. J Biomater Appl 2023;38:500–8. 37620997 10.1177/08853282231199313

[181] Dziadek M , Dziadek K, Checinska K, Zagrajczuk B, Cholewa-Kowalska K. Bioactive glasses modulate anticancer activity and other polyphenol-related properties of polyphenol-loaded PCL/bioactive glass composites. ACS Appl Mater Interfaces 2024;16:24261–73. 38709741 10.1021/acsami.4c02418 PMC11103658

[182] Sreeja S , Parameshwar R, Varma PRH, Sailaja GS. Hierarchically porous osteoinductive poly(hydroxyethyl methacrylate-co-methyl methacrylate) scaffold with sustained doxorubicin delivery for consolidated osteosarcoma treatment and bone defect repair. ACS Biomater Sci Eng 2021;7:701–17. 33395260 10.1021/acsbiomaterials.0c01628

[183] He P , Xu S, Guo Z, Yuan P, Liu Y, Chen Y, Zhang T, Que Y, Hu Y. Pharmacodynamics and pharmacokinetics of PLGA-based doxorubicin-loaded implants for tumor therapy. Drug Deliv 2022;29:478–88. 35147071 10.1080/10717544.2022.2032878 PMC8843208

[184] Cai JX , Liu JH, Wu JY, Li YJ, Qiu XH, Xu WJ, Xu P, Xiang DX. Hybrid cell membrane-functionalized biomimetic nanoparticles for targeted therapy of osteosarcoma. Int J Nanomedicine 2022;17:837–54. 35228800 10.2147/IJN.S346685 PMC8881933

[185] Deng J , Liu S, Li G, Zheng Y, Zhang W, Lin J, Yu F, Weng J, Liu P, Zeng H. pH-sensitive charge-conversion cinnamaldehyde polymeric prodrug micelles for effective targeted chemotherapy of osteosarcoma in vitro. Front Chem 2023;11:1190596. 37206197 10.3389/fchem.2023.1190596 PMC10188981

[186] Wang Z , Nogueira LP, Haugen HJ, Van Der Geest IC, de Almeida Rodrigues PC, Janssen D, Bitter T, van den Beucken J, Leeuwenburgh SC. Dual-functional porous and cisplatin-loaded polymethylmethacrylate cement for reconstruction of load-bearing bone defect kills bone tumor cells. Bioact Mater 2022;15:120–30. 35386344 10.1016/j.bioactmat.2021.12.023 PMC8941180

[187] Meng Z , Liu Y, Xu K, Sun X, Yu Q, Wu Z, Zhao Z. Biomimetic polydopamine-modified silk fibroin/curcumin nanofibrous scaffolds for chemo-photothermal therapy of bone tumor. ACS Omega 2021;6:22213–23. 34497912 10.1021/acsomega.1c02903 PMC8412900

[188] Gupta C , Hazra C, Poddar P, Dhara D, Byram PK, Chakravorty N, Sen R, Ghosh SK. Development and performance evaluation of self-assembled pH-responsive curcumin-bacterial exopolysaccharide micellar conjugates as bioactive delivery system. Int J Biol Macromol 2024;263:130372. 38395275 10.1016/j.ijbiomac.2024.130372

[189] Chen J , Qian C, Ren P, Yu H, Kong X, Huang C, Luo H, Chen G. Light-responsive micelles loaded with doxorubicin for osteosarcoma suppression. Front Pharmacol 2021;12:679610. 34220512 10.3389/fphar.2021.679610 PMC8249570

[190] Vijayakumar S , González-Sánchez ZI, Amanullah M, Sonamuthu J, Rajkumar M, Divya M, Durán-Lara EF, Li M. Shark chondroitin sulfate gold nanoparticles: a biocompatible apoptotic agent for osteosarcoma. Int J Biol Macromol 2025;290:138793. 39689798 10.1016/j.ijbiomac.2024.138793

[191] Liu P , Zhang W, Deng J, Zheng Y, Weng J, Yu F, Wang D, Zheng M, Kang B, Zeng H. Chain-shattering polymeric sulfur dioxide prodrug micelles for redox-triggered gas therapy of osteosarcoma. J Mater Chem B 2022;10:5263–71. 35762903 10.1039/d2tb00287f

[192] Malash AE , Al-Esnawy AA, Ereiba KT, Bakr AM, Abdraboh AS. An in vitro study of the effects of methotrexate loaded biocomposite beads on MG63 osteoblast cells. Sci Rep 2025;15:2231. 39825078 10.1038/s41598-025-85702-y PMC11748677

[193] Han R , Min Y, Li G, Chen S, Xie M, Zhao Z. Supercritical CO_2_-assisted fabrication of CM-PDA/SF/nHA nanofibrous scaffolds for bone regeneration and chemo-photothermal therapy against osteosarcoma. Biomater Sci 2023;11:5218–31. 37338001 10.1039/d3bm00532a

[194] Mushtaq A , Li L, Grøndahl L, Anitha A. Targeted nanoparticles based on alendronate polyethylene glycol conjugated chitosan for the delivery of siRNA and curcumin for bone metastasized breast cancer applications. Macromol Biosci 2024;24:e2300268. 37794635 10.1002/mabi.202300268

[195] Crisafulli E , Scalzone A, Tonda-Turo C, Girón-Hernández J, Gentile P. Multimodal layer-by-layer nanoparticles: a breakthrough in gene and drug delivery for osteosarcoma. J Mater Chem B 2024;12:12540–52. 39498896 10.1039/d4tb01541j

[196] Freeman FE , Dosta P, Shanley LC, Ramirez Tamez N, Riojas Javelly CJ, Mahon OR, Kelly DJ, Artzi N. Localized nanoparticle-mediated delivery of miR-29b normalizes the dysregulation of bone homeostasis caused by osteosarcoma whilst simultaneously inhibiting tumor growth. Adv Mater 2023;35:e2207877. 36994935 10.1002/adma.202207877

[197] Espona-Noguera A , Tampieri F, Canal C. Engineering alginate-based injectable hydrogels combined with bioactive polymers for targeted plasma-derived oxidative stress delivery in osteosarcoma therapy. Int J Biol Macromol 2024;257:128841. 38104678 10.1016/j.ijbiomac.2023.128841

[198] Zhang B , Zhang C, Chen C, Hong R, Shen Y, Yao C, Sun J, Zhang Y. Hybrid membranes-mediated biomimetic-nanoparticle carrying miR-665 for effective tumor treatment by remodeling tumor microenvironment. Int J Pharm 2025;675:125479. 40090634 10.1016/j.ijpharm.2025.125479

[199] Chu X , Mi B, Xiong Y, Wang R, Liu T, Hu L, Yan C, Zeng R, Lin J, Fu H, Liu G, Zhang K, Bian L. Bioactive nanocomposite hydrogel enhances postoperative immunotherapy and bone reconstruction for osteosarcoma treatment. Biomaterials 2025;312:122714. 39079462 10.1016/j.biomaterials.2024.122714

[200] Guo R , Zhang P, Liu J, Xie R, Wang L, Cai L, Qiu X, Sang H. NIR responsive injectable nanocomposite thermogel system against osteosarcoma recurrence. Macromol Rapid Commun 2022;43:e2200255. 35587472 10.1002/marc.202200255

[201] Francis AP , Augustus AR, Chandramohan S, Bhat SA, Priya VV, Rajagopalan R. A review on biomaterials-based scaffold: an emerging tool for bone tissue engineering. Mater Today Commun 2023;34:105124.

[202] Jiang S , Wang M, He J. A review of biomimetic scaffolds for bone regeneration: toward a cell-free strategy. Bioeng Transl Med 2021;6:e10206. 34027093 10.1002/btm2.10206 PMC8126827

[203] Chen X , Yang L, Wu Y, Wang L, Li H. Advances in the application of photothermal composite scaffolds for osteosarcoma ablation and bone regeneration. ACS Omega 2023;8:46362–75. 38107965 10.1021/acsomega.3c06944 PMC10720008

[204] Wang L , Yuan L, Dong Y, Huang W, Zhu J, Du X, Zhang C, Liu P, Mo J, Li B, Liu Z, Yu X, Yu H. Multifunctional 3D matrixes based on flexible bioglass nanofibers for potential application in postoperative therapy of osteosarcoma. Regen Biomater 2024;11:rbae088. 39165883 10.1093/rb/rbae088 PMC11333569

[205] Percival KM , Paul V, Husseini GA. Recent advancements in bone tissue engineering: integrating smart scaffold technologies and bio-responsive systems for enhanced regeneration. Int J Mol Sci 2024;25:6012. 38892199 10.3390/ijms25116012 PMC11172494

[206] Konovalenko VF , Ternovyi NK, Tuz EV, Protsenko VV, Solonitsyn EO, Audai A, Drobotun OV, Ulianchych NV. Experimental substantiation of the use of hydroxyapatite–tricalcium phosphate bioceramics for replacing bone defects after tumor removal. Exp Oncol 2021;43:237–41. 34591421 10.32471/exp-oncology.2312-8852.vol-43-no-3.16584

[207] Nayak AK , Maity M, Barik H, Behera SS, Dhara AK, Hasnain MS. Bioceramic materials in bone-implantable drug delivery systems: a review. J Drug Deliv Sci Technol 2024;95:105524.

[208] Barreto MEV , Medeiros RP, Shearer A, Fook MVL, Montazerian M, Mauro JC. Gelatin and bioactive glass composites for tissue engineering: a review. J Funct Biomater 2022;14:23. 36662070 10.3390/jfb14010023 PMC9861949

[209] Houaoui A , Szczodra A, Lallukka M, El-Guermah L, Agniel R, Pauthe E, Massera J, Boissiere M. New generation of hybrid materials based on gelatin and bioactive glass particles for bone tissue regeneration. Biomolecules 2021;11:444. 33802745 10.3390/biom11030444 PMC8002581

[210] Souza L , Ferreira FV, Lopes JH, Camilli JA, Martin RA. Cancer inhibition and in vivo osteointegration and compatibility of gallium-doped bioactive glasses for osteosarcoma applications. ACS Appl Mater Interfaces 2022;14:45156–66. 36170227 10.1021/acsami.2c12102 PMC9562271

[211] Foroughi AH , Valeri C, Razavi MJ. A review of computational optimization of bone scaffold architecture: methods, challenges, and perspectives. Prog Biomed Eng 2025;7:012003. 10.1088/2516-1091/ad879a 39655853

[212] Pektas HK , Demidov Y, Ahvan A, Abie N, Georgieva VS, Chen S, Farè S, Brachvogel B, Mathur S, Maleki H. MXene-integrated silk fibroin-based self-assembly-driven 3D-printed theragenerative scaffolds for remotely photothermal anti-osteosarcoma ablation and bone regeneration. ACS Mater Au 2023;3:711–26. 38089660 10.1021/acsmaterialsau.3c00040 PMC10636780

[213] Geng Y , Liu T, Zhao M, Wei H, Yao X, Zhang Y. Silk fibroin/polyacrylamide-based tough 3D printing scaffold with strain sensing ability and chondrogenic activity. Compos B: Eng 2024;271:111173.

[214] Shao R , Dong Y, Zhang S, Wu X, Huang X, Sun B, Zeng B, Xu F, Liang W. State of the art of bone biomaterials and their interactions with stem cells: current state and future directions. Biotechnol J 2022;17:e2100074. 35073451 10.1002/biot.202100074

[215] Shang F , Yu Y, Liu S, Ming L, Zhang Y, Zhou Z, Zhao J, Jin Y. Advancing application of mesenchymal stem cell-based bone tissue regeneration. Bioact Mater 2021;6:666–83. 33005830 10.1016/j.bioactmat.2020.08.014 PMC7509590

[216] Liang J , Liu P, Yang X, Liu L, Zhang Y, Wang Q, Zhao H. Biomaterial-based scaffolds in promotion of cartilage regeneration: recent advances and emerging applications. J Orthop Translat 2023;41:54–62. 37691640 10.1016/j.jot.2023.08.006 PMC10485599

[217] Hong I-S. Enhancing stem cell-based therapeutic potential by combining various bioengineering technologies. Front Cell Dev Biol 2022;10:901661. 35865629 10.3389/fcell.2022.901661 PMC9294278

[218] Sharma S , Muthu S, Jeyaraman M, Ranjan R, Jha SK. Translational products of adipose tissue-derived mesenchymal stem cells: bench to bedside applications. World J Stem Cells 2021;13:1360–81. 34786149 10.4252/wjsc.v13.i10.1360 PMC8567449

[219] Lau CS , Park SY, Ethiraj LP, Singh P, Raj G, Quek J, Prasadh S, Choo Y, Goh BT. Role of adipose-derived mesenchymal stem cells in bone regeneration. Int J Mol Sci 2024;25:6805. 38928517 10.3390/ijms25126805 PMC11204188

[220] Gallo MC , Elias A, Reynolds J, Ball JR, Lieberman JR. Regional gene therapy for bone tissue engineering: a current concepts review. Bioengineering 2025;12:120. 40001640 10.3390/bioengineering12020120 PMC11852166

[221] Qin L , Yang S, Zhao C, Yang J, Li F, Xu Z, Yang Y, Zhou H, Li K, Xiong C, Huang W, Hu N, Hu X. Prospects and challenges for the application of tissue engineering technologies in the treatment of bone infections. Bone Res 2024;12:28. 38744863 10.1038/s41413-024-00332-w PMC11094017

[222] Ren S , Wang M, Wang C, Wang Y, Sun C, Zeng Z, Cui H, Zhao X. Application of non-viral vectors in drug delivery and gene therapy. Polymers 2021;13:3307. 34641123 10.3390/polym13193307 PMC8512075

[223] Laird NZ , Acri TM, Tingle K, Salem AK. Gene- and RNAi-activated scaffolds for bone tissue engineering: current progress and future directions. Adv Drug Deliv Rev 2021;174:613–27. 34015421 10.1016/j.addr.2021.05.009 PMC8217358

[224] Luo R , Le H, Wu Q, Gong C. Nanoplatform-based in vivo gene delivery systems for cancer therapy. Small 2024;20:e2312153. 38441386 10.1002/smll.202312153

[225] Kim B , Pradhan L, Hernandez A, Yenurkar D, Nethi SK, Mukherjee S. Current advances in immunomodulatory biomaterials for cell therapy and tissue engineering. Adv Therap 2023;6:2300002.

[226] Nakkala JR , Li Z, Ahmad W, Wang K, Gao C. Immunomodulatory biomaterials and their application in therapies for chronic inflammation-related diseases. Acta Biomater 2021;123:1–30. 33484912 10.1016/j.actbio.2021.01.025

[227] Batool F , Özçelik H, Stutz C, Gegout P-Y, Benkirane-Jessel N, Petit C, Huck O. Modulation of immune-inflammatory responses through surface modifications of biomaterials to promote bone healing and regeneration. J Tissue Eng 2021;12:20417314211041428. 34721831 10.1177/20417314211041428 PMC8554547

[228] Thanigaivel S , Priya AK, Balakrishnan D, Dutta K, Rajendran S, Soto-Moscoso M. Insight on recent development in metallic biomaterials: strategies involving synthesis, types and surface modification for advanced therapeutic and biomedical applications. Biochem Eng J 2022;187:108522.

[229] Wang X , Wang L, Hao Q, Cai M, Wang X, An W. Harnessing glucose metabolism with nanomedicine for cancer treatment. Theranostics 2024;14:6831–82. 39479443 10.7150/thno.100036 PMC11519798

[230] Cheng Q , Li R, He Y, Zhu Y, Kang Y, Ji X. Genetically engineered cellular nanovesicles: theories, design and perspective. Adv Funct Mater 2024;34:2407842.

[231] Deshmukh R , Sethi P, Singh B, Shiekmydeen J, Salave S, Patel RJ, Ali N, Rashid S, Elossaily GM, Kumar A. Recent review on biological barriers and host–material interfaces in precision drug delivery: advancement in biomaterial engineering for better treatment therapies. Pharmaceutics 2024;16:1076. 39204421 10.3390/pharmaceutics16081076 PMC11360117

[232] Damiri F , Fatimi A, Santos ACP, Varma RS, Berrada M. Smart stimuli-responsive polysaccharide nanohydrogels for drug delivery: a review. J Mater Chem B 2023;11:10538–65. 37909361 10.1039/d3tb01712e

[233] Gong Z , Peng S, Cao J, Tan H, Zhao H, Bai J. Advances in the variations and biomedical applications of stimuli-responsive nanodrug delivery systems. Nanotechnology 2024;35:132001. 10.1088/1361-6528/ad170b 38198449

[234] Poh PSP , Lingner T, Kalkhof S, Märdian S, Baumbach J, Dondl P, Duda GN, Checa S. Enabling technologies towards personalization of scaffolds for large bone defect regeneration. Curr Opin Biotechnol 2022;74:263–70. 35007988 10.1016/j.copbio.2021.12.002

[235] Luo Y. Toward fully automated personalized orthopedic treatments: innovations and interdisciplinary gaps. Bioengineering 2024;11:817. 39199775 10.3390/bioengineering11080817 PMC11351140

[236] Wei X , Tang Z, Wu H, Zuo X, Dong H, Tan L, Wang W, Liu Y, Wu Z, Shi L, Wang N, Li X, Xiao X, Guo Z. Biofunctional magnesium-coated Ti6Al4V scaffolds promote autophagy-dependent apoptosis in osteosarcoma by activating the AMPK/mTOR/ULK1 signaling pathway. Mater Today Bio 2021;12:100147. 10.1016/j.mtbio.2021.100147 PMC8523865 34704011

[237] Hui T , Fu J, Zheng B, Fu C, Zhao B, Zhang T, Zhang Y, Wang C, Yu L, Yang Y, Yue B, Qiu M. Subtractive nanopore engineered MXene photonic nanomedicine with enhanced capability of photothermia and drug delivery for synergistic treatment of osteosarcoma. ACS Appl Mater Interfaces 2023;15:50002–14. 37851535 10.1021/acsami.3c10572

[238] Shahed CA , Ahmad F, Günister E, Foudzi FM, Ali S, Malik K, Harun WSW. Antibacterial mechanism with consequent cytotoxicity of different reinforcements in biodegradable magnesium and zinc alloys: a review. J Magnes Alloys 2023;11:3038–58.

[239] Wang D , Peng Y, Li Y, Kpegah JKSK, Chen S. Multifunctional inorganic biomaterials: new weapons targeting osteosarcoma. Front Mol Biosci 2023;9:1105540. 36660426 10.3389/fmolb.2022.1105540 PMC9846365

